# Polymer Membranes for All-Vanadium Redox Flow Batteries: A Review

**DOI:** 10.3390/membranes11030214

**Published:** 2021-03-18

**Authors:** Dennis Düerkop, Hartmut Widdecke, Carsten Schilde, Ulrich Kunz, Achim Schmiemann

**Affiliations:** 1 Institute of Recycling, Ostfalia University of Applied Sciences, Robert-Koch-Platz 8a, 38440 Wolfsburg, Germany; h.widdecke@ostfalia.de (H.W.); a.schmiemann@ostfalia.de (A.S.); 2 Institute of Particle Technology, Braunschweig University of Technology, Volkmaroder Straße 5, 38100 Braunschweig, Germany; c.schilde@tu-bs.de; 3 Institute of Chemical and Electrochemical Process Engineering, Clausthal University of Technology, Leibnizstraße 17, 38678 Clausthal-Zellerfeld, Germany; kunz@icvt.tu-clausthal.de

**Keywords:** all-vanadium redox flow battery, polymer membrane, efficiency, current density, costs

## Abstract

Redox flow batteries such as the all-vanadium redox flow battery (VRFB) are a technical solution for storing fluctuating renewable energies on a large scale. The optimization of cells regarding performance, cycle stability as well as cost reduction are the main areas of research which aim to enable more environmentally friendly energy conversion, especially for stationary applications. As a critical component of the electrochemical cell, the membrane influences battery performance, cycle stability, initial investment and maintenance costs. This review provides an overview about flow-battery targeted membranes in the past years (1995–2020). More than 200 membrane samples are sorted into fluoro-carbons, hydro-carbons or N-heterocycles according to the basic polymer used. Furthermore, the common description in membrane technology regarding the membrane structure is applied, whereby the samples are categorized as dense homogeneous, dense heterogeneous, symmetrical or asymmetrically porous. Moreover, these properties as well as the efficiencies achieved from VRFB cycling tests are discussed, e.g., membrane samples of fluoro-carbons, hydro-carbons and N-heterocycles as a function of current density. Membrane properties taken into consideration include membrane thickness, ion-exchange capacity, water uptake and vanadium-ion diffusion. The data on cycle stability and costs of commercial membranes, as well as membrane developments, are compared. Overall, this investigation shows that dense anion-exchange membranes (AEM) and N-heterocycle-based membranes, especially poly(benzimidazole) (PBI) membranes, are suitable for VRFB requiring low self-discharge. Symmetric and asymmetric porous membranes, as well as cation-exchange membranes (CEM) enable VRFB operation at high current densities. Amphoteric ion-exchange membranes (AIEM) and dense heterogeneous CEM are the choice for operation mode with the highest energy efficiency.



**Table of Contents**
1.Introduction      22.Commercial membranes for VRFB Work      53.Polymer membrane Development      9
3.1 Membrane Structures      9
3.2 Membrane Polymers      104. Polymer membrane Development      11
4.1 Membrane Properties      15
4.2 Membrane Impact on VRFB Cell Performance      325. Cycle Stability      466. Membrane Costs      477. Conclusions      488. Acknowledgements      509. Abbreviations      5110. References      52


## 1. Introduction

The growing use of renewable energy supplements fossil fuel and nuclear power in many parts of the world with considerable proportions [1,2]. The expansion of the electrical grid and the efficient use of fluctuating energies still pose challenges, which are fundamentally associated with costs. In addition to lithium-ion batteries, flow-batteries have increasingly gained interest. Redox flow batteries have external tanks to store electric energy in vanadium-based electrolytes. The electrolytes are pumped through the battery stack for energy conversion (charging or discharging). This is the main advantage of flow batteries. The power depends on the stack size and the capacity on the volume of the tanks.

**Figure 1:**
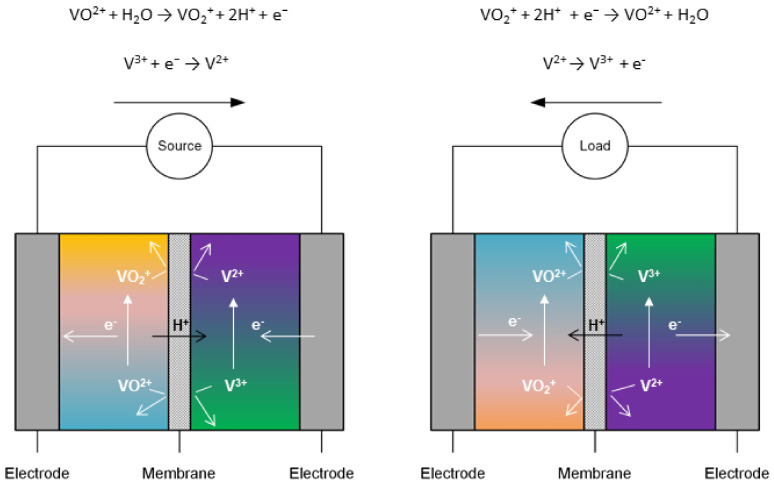
Schematic of the all-Vanadium redox flow battery (**left** charging/**right** discharging).

The intermediate storage of electrical energy in all-vanadium redox flow batteries (Figure 1) is being pursued seriously and is demonstrated in numerous pilot projects and industrial installations (e.g., cellcube [3], Rongke Power [4], Sumitomo Electric [5], Fraunhofer ICT [6]). These highly efficient electrochemical storage units can generally be industrially manufactured in large quantities [7,8,9]. Its intended use can be either as part of large-scale plants or for consumer use in smaller applications. Studies on techno-economic assessment of VRFB are analyzed in [10]. As a result, guide values of 650 EUR (kWh)^−1^ and 550 EUR (kWh)^−1^ for VRFB systems in a power range of 10–1000 kW providing electrical energy for 4 h and 8h are derived from literature. Here, the key components of the electrochemical cell, the active species vanadium, the membranes, the electrode felts and bipolar plates differ in proportion to the total system costs. The proportion of Vanadium costs, membrane costs and electrode felt/bipolar plate costs to total system costs is about 30–60%, 3–30% and below 5%.

Cost reduction is one way to realize marketable products. As an integral part of the VRFB, the membrane influences investment costs, service life and battery performance. Perfluorosulfonic acid (PFSA) membranes account for about 40% of the investment costs of a VRFB stack. Like other VRFB components, the membrane influences the efficiency and power of the cells. Looking at membrane development as a way to optimize costs, these could be reduced by making cheaper membranes available on the market. The costs consist of the specific cost of raw materials, the manufacturing process and, in particular, on the quantity produced.

Generally, high proton conductivity and high H^+^/V selectivity are the main issues to overcome in designing membranes for VRFB. Mechanical and chemical stability (resistance to the highly oxidizing electrolyte of the positive half-cell) during VRFB operation are the main issues membrane materials have to overcome.

The development of membrane materials for VRFB has been an ongoing process for decades. From 2011 to 2020, several review papers were published summarizing the most important membrane developments. In [11] Li et al. describe the basic properties of VRFB and its development history. The first demonstration projects are mentioned and the electrical performances and storage capacities achieved are listed. For ion-exchange membranes, requirements and parameters relevant for the operation of the VRFB are described. The production of membranes is also briefly discussed. Furthermore, membrane developments published over a period of about 10 years are listed, differentiating between the various material groups: pore filled membranes; perfluorinated membranes; modified perfluorinated membranes; partially fluorinated IEMs and non-fluorinated membranes. In [12], an overview of membrane properties that are relevant for the VRFB is given. Furthermore, membrane types that can be used in the VRFB are summarized. They are divided into cation-exchange membranes, anion-exchange membranes, amphoteric membranes and non-ionic membranes. H. Prifti et al. [13] provide a brief introduction to the design, manufacture and characterization of ion-exchange membranes. The modification of membranes to improve VRFB performance is discussed and various examples are given. H. H. Cha [14] summarizes the efforts to develop nanocomposite membranes for VRFB. The developments focus on the reduction of vanadium-ion permeability, the improvement of proton conductivity for improved battery performance and a long service life of the battery systems. The focus is on functionalized materials for nanocomposite membranes. The description of membrane properties and the calculation of the coulombic, voltage and energy efficiency are described in detail in previous reviews [11,13,15,16] and are not discussed further here. Table 1 gives an overview of published review papers considering important membrane properties and membranes for redox flow batteries.

Publications of new membranes and their respective tests in VRFB cells have increased significantly since 2012. In addition to the information provided in [11,12,13,14] further developments have been added to the range of membrane types described above. In this review, the basic classification of the membranes into fluoro-carbon, hydro-carbon and N-heterocycle-based membranes is made. They are additionally classified according to their structure and indicated by their chemical character as CEM, AEM, AIEM or non-ionic.

The main component of this review is a digest of relevant research results from previous years, too. Thereby, the focus is on membrane main polymers and the impact of new membranes on VRFB performances. Especially, the graphical presentation of the data should provide a comprehensive overview of achievable performance for VRFB cells using different membranes. Knowing that the performance of a VRFB cell is not exclusively dependent on the membrane used, the results are displayed next to each other in order to provide a simplified overview of different membrane types.

## 2. Commercial Membranes for VRFB

**Figure 2:**
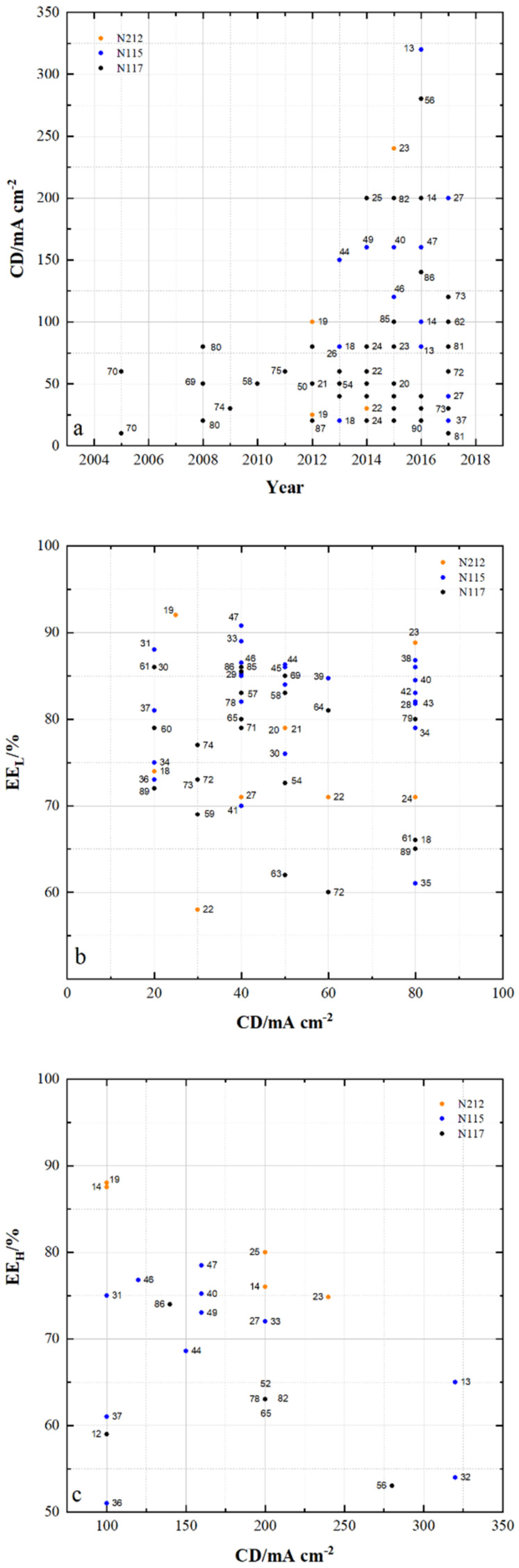
Results from VRFB tests using Nafion: (**a**) current density during cycling tests (**b**) energy efficiency of VRFB cells at current densities < 100 mA cm^−^^2^ and (**c**) energy efficiency of VRFB cells at current densities ≥ 100 mA cm^−2^

Various companies offer ion-exchange membranes, shown in Table 2, for use in VRFB. Here, the coulombic, voltage and energy efficiencies (CE, VE and EE) are indicated for the respective current densities (CD). These membranes differ in their application for different operating modes, which are divided into energy efficient operation, operation with high current densities and operation with low self-discharge [26]. The “Vanadion” membrane was developed on the basis of Nafion. A thin, more selective Nafion layer was applied to a microporous layer. The membrane is 230 µm thick and provides a constant energy efficiency over 80 charge/discharge cycles [27]. The Nafion membranes N212, N115 and N117 have been commercially available for many years and are often used as reference membranes in comparison to newly developed membrane samples (MS). The measurement results from many studies regarding VRFB efficiencies can be found in Table 3 and Figure 2. More than two decades ago Asahi Glass Co. Ltd. has developed hydro-carbon-based anion exchange membranes with improved chemical stability in VRFB [28,29]. This resulted in the oxidation-stabilized AEM Selemion™ APS4 membrane, which is distributed by AGC [30]. Solvay offered the short side chain PFSA membrane known as Aquivion, which was successfully tested in VRFB [31]. It should be noted that due to different test conditions, the respective efficiency results for the membranes used cannot be compared directly with each other.

In VRFB cells, Nafion is suitable as a reference membrane due to its known properties in electrochemical cells and its worldwide availability. Nafion, for example, is preferably used to show the change in battery performance caused by changing the membrane component. Table 3 lists efficiencies measured with different Nafion types as reference membranes for comparison measurements in VRFB cells. N212, N115 and N117 were used in the cycling tests at different current densities. B. Jang et al. [36] describe the influence of Nafion membrane pretreatment on battery performance.

The tabular listing of the results shows how efficiencies under different test conditions vary. The lowest energy efficiency of 51% is achieved with N115 (MS34) and the highest energy efficiency of 92% with N212 (MS19).

Figure 2a shows the current density used in VRFB cycling experiments with Nafion membranes and the year in which the measurements were published. The numbers close to the data points refer to the sample numbers of the membranes in Table 3. It can be seen that from 2012 onwards cycling tests could increasingly be carried out at current densities greater than 100 mA cm^−2^. With N212 tests up to a current density of 240 mA cm^−2^, with N115 tests with a current density up to 320 mA cm^-2^ and with N117 tests with a current density up to 260 mA cm^−2^ were performed. This means that progress has been made through cell development in recent years and the research potential does not yet seem to be exhausted.

Figure 2b shows the energy efficiency achieved using Nafion membranes at current densities below 100 mA cm^−2^. The differing VRFB cells and varied test conditions lead to the specific results for each membrane used at the respective current density. For example, energy efficiencies between 66% and 89% could be achieved for the current density of 80 mA cm^−2^ using N212 membranes, between 61% and 87% for N115 membranes and between 65% and 80% for N117 membranes. At current densities between 20 and 40 mA cm^−2^, efficiencies of over 90% were measured with N212 and N115.

Figure 2c shows the energy efficiencies at current densities of at least 100 mA cm^−2^ for N212, N115 and N117. While with current densities below 100 mA cm^−2^ there is no correlation between current density and energy efficiency, with current densities of at least 100 mA cm^−2^ there is a tendency of decreasing efficiency with increasing current density up to above 200 mA cm^−2^ for all three membranes N212, N115 and N117. Furthermore, the highest energy efficiencies when using the respective membrane at 100 and 200 mA cm^−2^ show the increase in efficiency when the membrane thickness decreases:

EE (N212) > EE (N115) > EE (N117).

Figure 2 clearly shows that the energy efficiency of VRFB cells is related to the membrane used. However, differences in efficiency cannot be exclusively allocated to the specific membrane used due to different cell designs and operation modes of the cells.

## 3. Polymer Membrane Development

Synthetic membranes are used in various processes such as reverse osmosis, water filtration, dialysis or electrolysis. The respective separation requires membranes with a certain structure and certain chemical properties which result from the specific manufacturing process, formulation conditions and materials used. In electrochemical cells, electrical properties of membranes (membrane/electrolyte) and selectivity are particularly important. VRFB research focuses mainly on polymer membranes due to the generally low material and manufacturing costs involved when produced in large quantities.

### 3.1. Membrane Structures

**Figure 3:**
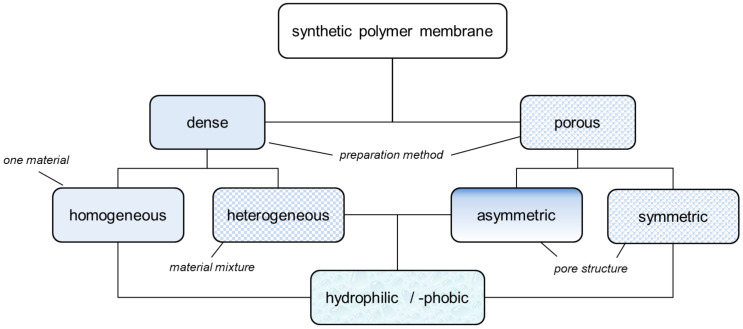
Classification of synthetic membranes according to their structure.

Membrane properties not only depend on materials chosen but also on the manufacturing process. The general classification according to their structure and material is shown in Figure 3 [106,107,108]. Whereas dense membranes can be obtained by polymer extrusion or phase inversion by solvent evaporation (e.g., doctor blading) [106,109], porous membranes and separators are made by “stretching” semicrystalline polymer films [106,108]. Other known processes for the production of porous membranes are the sintering of polymer powders, thermal-induced phase separation or diffusion-induced phase separation. Ion-exchange membranes, which are a type of dense membrane, are produced by phase inversion, by solvent evaporation or special polymerization processes with corresponding chemical post-treatment [110].

While several materials are required to prepare a heterogeneous dense membrane, e.g., composite or multilayer membrane, only one polymer or a polymer blend is typically used for homogeneous dense and symmetrically porous membranes. Asymmetrically porous membranes can be prepared from one (integral-asymmetrical) or several polymers (composite-asymmetrical) [107]. In the production of integrally asymmetrically porous membranes, first a thin skin is created on a polymer solution by phase inversion through solvent evaporation. Following this, the remaining solvent is extracted from the polymer solution by immersion in a precipitation bath. With asymmetrical composite membranes, a thin layer (dense or reduced porosity) is applied to a symmetrically porous membrane [107].

Ion-exchange membranes such as Nafion and symmetrically porous membranes such as Daramic have been tested in VRFB for many years. The chemical modification of Daramic by applying charge carriers has resulted in an improvement of the energy efficiency [111]. In this review the membrane structures of published membrane samples are divided into homogeneous dense (dho), heterogeneous dense (dhe), symmetrically porous (sym) and asymmetrically porous (asym) membranes.

### 3.2. Membrane Polymers

In this review, modified polymer membranes for VRFB are presented from more than 190 publications up to and including the year 2020. In most cases, the developments are based on polymers that can already be produced on a large scale. These include poly(ether ether ketone) (PEEK), poly(sulfone) (PSU), poly(ether sulfone) (PES), poly(phenyl sulfone) (PPSU), poly(vinylidene fluoride) (PVDF), poly(ethylene-tetrafluoroethylene) (ETFE), poly(benzimidazole) (PBI), poly(imide) (PI), perfluorosulfonic acid (PFSA), poly(phenylene ether) (PPE) and poly(tetrafluoroethylene) (PTFE). Other modified membrane polymers investigated include fluorinated poly(arylene ether) (FPAE), poly(fluorenyl ether ketone) (PF), poly(phenylene) (PPh) and poly(phthalazinone ether ketone) (PPEK).

For a clear presentation of the membrane developments, these are summarized according to the most important structural feature (C-F, ether-ketones, ether-sulfones, fluorenyles, phenylenes, benzimidazoles, phthalazinone-ether and imides) of the monomer (Table 4).

Cation-exchange membranes (CEM), anion-exchange membranes (AEM) and amphoteric ion-exchange membranes (AIEM) are based on polymers with covalently bonded charges. AEM* and AIEM* are based on neutral polymers which build up a positive charge through interaction with hydronium-ions by lowering the pH value in their environment below pH 7.

## 4. Membrane Developments in Recent Years

The number of publications concerning membrane samples for VRFB has increased significantly since 2012. Polymer membranes can be subdivided with regard to elementary components of the polymer main chains. A distinction is made between fluorine-based polymers (fluoro-carbon) and fluorine-free (hydro-carbon) polymers. Nitrogen containing heterocycles-based polymers (*N*-heterocycles) are also used.

**Figure 4:**
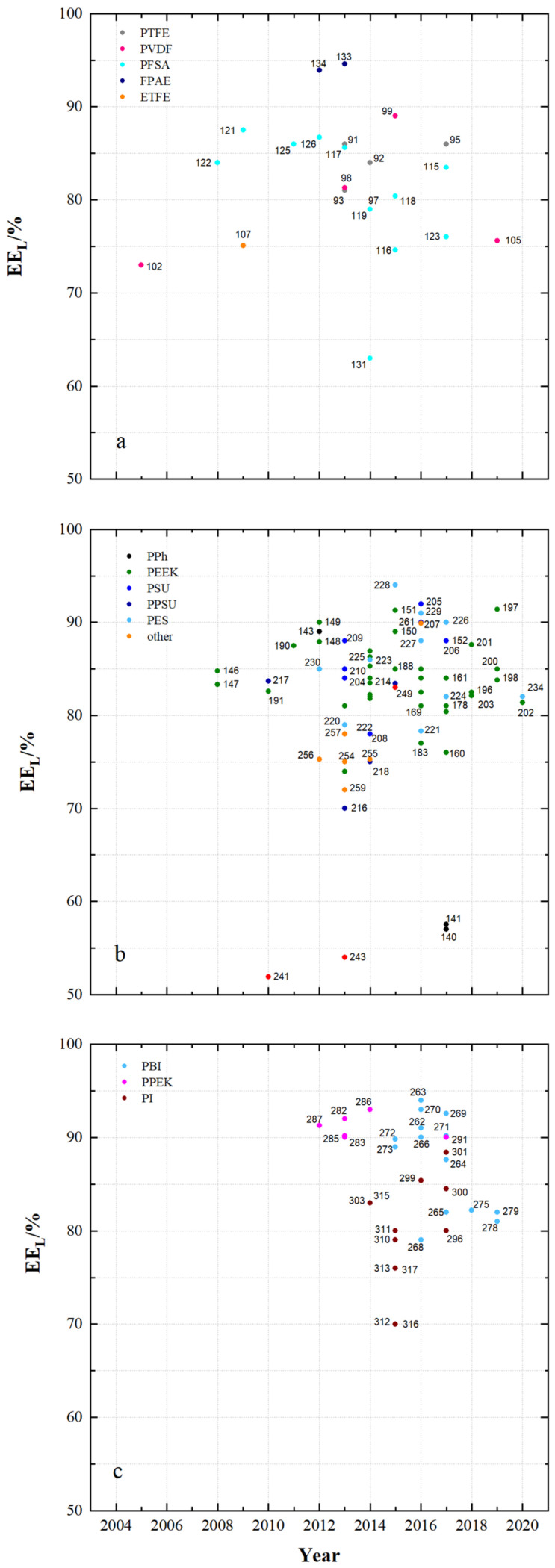
The energy efficiency of VRFB cells at current densities < 100 mA cm^−^^2^ using: (**a**) fluoro-carbons, (**b**) hydro-carbons and (**c**) *N*-heterocycles.

For membranes composed of polymer mixtures, this subdivision refers to the polymer with the larger molar or mass fraction in the mixture. Polymers from these three groups are used in the development of polymer membranes for VRFB. In order to optimize the performance or the costs of a VRFB cell, suitable methods are selected to make chemical modifications to the polymers or to generate desired spatial structures. These chemical modifications can be applied to commercially available films (Figure 4a, MS107), commercial polymers (Figure 4b, MS151), or proprietary polymer syntheses (Figure 4b, MS143). The chemical methods aim to generate acidic, basic or amphoteric polymers whose chemical function influences the internal resistance of the VRFB and the cross-over between the half cells. As shown in Figure 3, synthetic membranes with distinctive structures can be used in VRFB cells.

The developed and published membrane samples of the past years show these different structures and were tested in VRFB. An exception is the dense hydrophobic membrane, which can be used as a separation medium for gases but would act as an insulator in a VRFB. Dense homogeneous membranes are composed of fluoro-carbons (e.g., PFSA, ETFE-g-X, PVDF-g-X, sFPAE), hydro-carbons (e.g., QDAPP, sPEEK, sPSU) and N-heterocycles (PBI, sPI).

**Figure 5:**
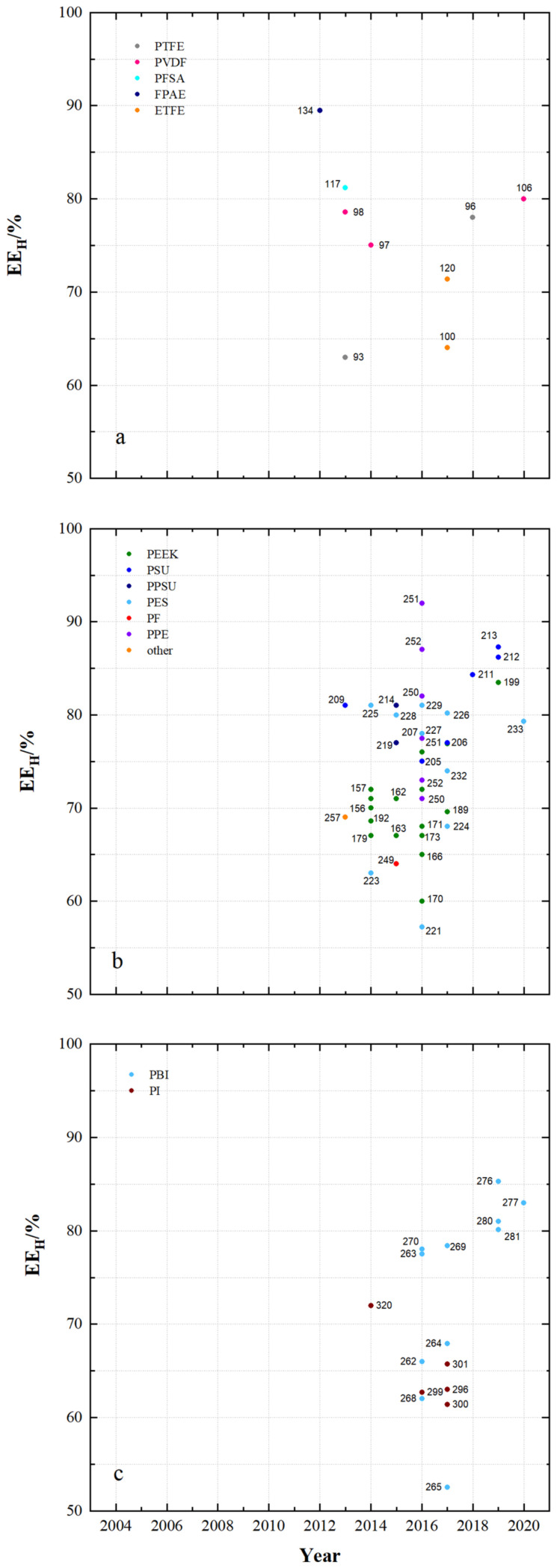
The energy efficiency of VRFB cells by using the developed membrane sample at current densities ≥ 100 mA cm^−2^ in recent years: (**a**) fluoro-carbons, (**b**) hydro-carbons and (**c**) *N*-heterocycles.

Dense heterogeneous membranes represent a very frequently used membrane type, because it is relatively easy to influence their properties such as porosity (Figure 5b, MS189) by adding disperse components. Dense heterogeneous membranes can be found in fluoro-carbon, hydro-carbon and *N*-heterocycle-based membranes.

Porous membranes can either have hydrophilic or hydrophobic character. Symmetrically porous (Figure 4c, MS266, hydrophilic) and asymmetrically porous (Figure 4a, MS98, hydrophobic) membranes get prepared and tested in scientific workgroups.

Furthermore, multi-layer membranes are constructed to achieve certain properties, such as improved chemical stability (Table 7, MS167), lower costs (Figure 4b, MS147) or lower cross-over (Figure 4a, MS95).

Figure 4 and Figure 5 show the observed energy efficiency of VRFB cells using the developed membrane samples and the publication year. Figure 4 shows results obtained with current densities less than 100 mA cm^−2^ and Figure 5 shows results obtained with current densities greater than 100 mA cm^−2^. The figures provide an overall picture of the achievable energy efficiencies of VRFB with different flow-battery targeted membranes.

When comparing the results of the commercial membranes in Figure 2b with the results in Figure 4, it can be observed that VRFB with new membrane developments often achieve higher energy efficiencies than VRFB with each reference membranes.

A direct comparison of membranes is only possible in the same cell and under identical experimental conditions. The comparison can be expressed in numerical terms by comparing the energy efficiencies achieved according to Equation (1). A value less than 1 is obtained if the energy efficiency of the VRFB using flow-battery targeted membranes is less than the energy efficiency of the VRFB with a reference membrane. EE_r_ (energy efficiency ratio) is larger than one if the energy efficiency of the VRFB using flow-battery targeted membranes is higher. It should be mentioned that modifications to the cell other than the membrane further influence individual EE and thus the resulting ratio EE_r_.

(1) $${\mathrm{EE}}_{r}=\frac{{\mathrm{EE}}_{1}}{{\mathrm{EE}}_{2}}$$

EE_1_ is the energy efficiency of the VRFB cell using flow-battery targeted membranes and EE_2_ the energy efficiency of the VRFB cell using a reference membrane. These energy efficiency ratios as well as the reference membranes are also given in Table 5, Table 6, Table 7, Table 8, Table 9, Table 10, Table 11, Table 12, Table 13 and Table 14.

While energy efficiency describes the performance of a VRFB by charge and discharge cycles, membrane characteristics are usually influenced by a number of parameters. The frequently investigated membrane properties which can influence VRFB performance are the membrane thickness, water uptake, ion-exchange capacity, electrical resistance and the diffusion coefficient for vanadyl-cations. Furthermore, the ion-selectivity [112] is an important quality. Equation (2), like Equation (1), calculates a ratio for comparison to the reference membrane.

(2) $$D_{r}=\frac{D_{c1}}{D_{c2}}$$

D_c1_ is the diffusion coefficient (VO^2+^) of the membrane samples and D_c2_ the diffusion coefficient (VO^2+^) of the reference membranes.

Table 5, Table 6, Table 7, Table 8, Table 9, Table 10, Table 11, Table 12, Table 13 and Table 14 list the best membrane sample selected from each publication. The tables contain information about the specific membrane sample as well as VRFB performance data. In addition to the sample name from the respective publication, the starting polymer, the structure and the chemical character (AEM, CEM or AIEM) are also listed.

### 4.1. Membrane Properties

**Figure 6:**
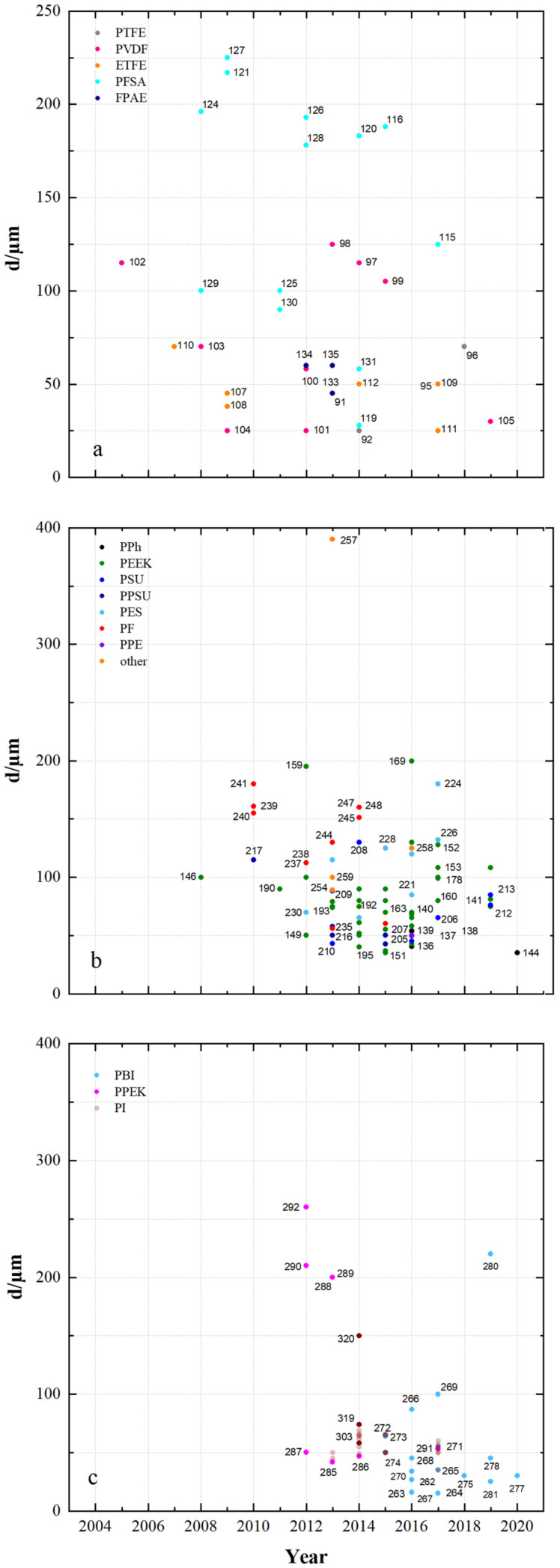
The thickness of developed membranes in recent years: (**a**) fluoro-carbons, (**b**) hydro-carbons and (**c**) *N*-heterocycles.

Membrane samples are produced by chemical modification of commercially available membranes and films or by coating and subsequent phase inversion (solvent evaporation or precipitation). The thickness of the membrane samples depicted in Figure 6 is the result from these processes and also of possibly occurring membrane swelling in battery electrolyte.

Generally, the thickness of the membrane defines the distance between the electrodes. For a low electric resistance of an electrochemical cell, the smallest distance between the electrodes is desirable.

The fluoro-carbon-based membranes (Figure 6a) have thicknesses from 25 µm to 225 µm, the hydro-carbon-based membranes in Figure 6b thicknesses from 35 µm to 390 µm and the *N*-heterocycle-based membranes in Figure 6c thicknesses from 15 µm to 260 µm.

The PFSA membranes MS116, MS120, MS121, MS124, MS126, MS127 and MS128 are modified Nafion (N117) membranes with an original thickness of about 180 µm.

ETFE membranes modified by grafting (MS107-MS114) use commercially available ETFE films (25 or 50 µm). A commercial film is also used for the pore-filled PTFE membrane (MS92).

The non-ionic PVDF membranes MS97, MS98 and MS99 have porous structures. The hydrophobic and asymmetrically porous MS98 has a separation layer in the submicron range with pore sizes of about 50 nm. The hydrophilic asymmetrically porous MS99 has a similar structure.

Further asymmetric membranes can be found in the group of hydro-carbon-based polymer membranes. These include MS208 with an average pore size of 1.78 nm, MS225 (asymmetrically composed) with a Nafion separating layer of approximately 1 µm and MS229 (asymmetrically composed) modified by a zeolite layer with pore sizes of 0.3–1 nm.

In addition to an asymmetrically porous membrane, MS269 with a separating layer of about 5 µm, symmetrically porous PBI-based membranes with thicknesses between 34 µm (MS270) and 87 µm (MS266) were developed.

The thickness of a membrane defines the distance between the electrodes in the VRFB and directly influences the material cost. Furthermore, separation effect increases with the thickness of the membrane up to a critical pore size.

The simultaneous reactions on the anode and cathode surfaces during charging and discharging require the exchange of protons. Sulfuric acid, the solvent of the reactive vanadium species, as well as sulfonated polymers are excellent proton conductors. The ion exchange capacity of a membrane describes the acid concentration of the polymer membrane. The ion-exchange capacities (IEC_c_, cations) indicated for PFSA membranes are between 0.85 and 1.67 mmol g^−1^, whereby MS129 containing sulfonated PEEK in addition to Nafion enables the highest IEC_c_. The FPAE membranes have an IEC_c_ of 1.6 to 1.8 mmol g^−1^.

**Figure 7:**
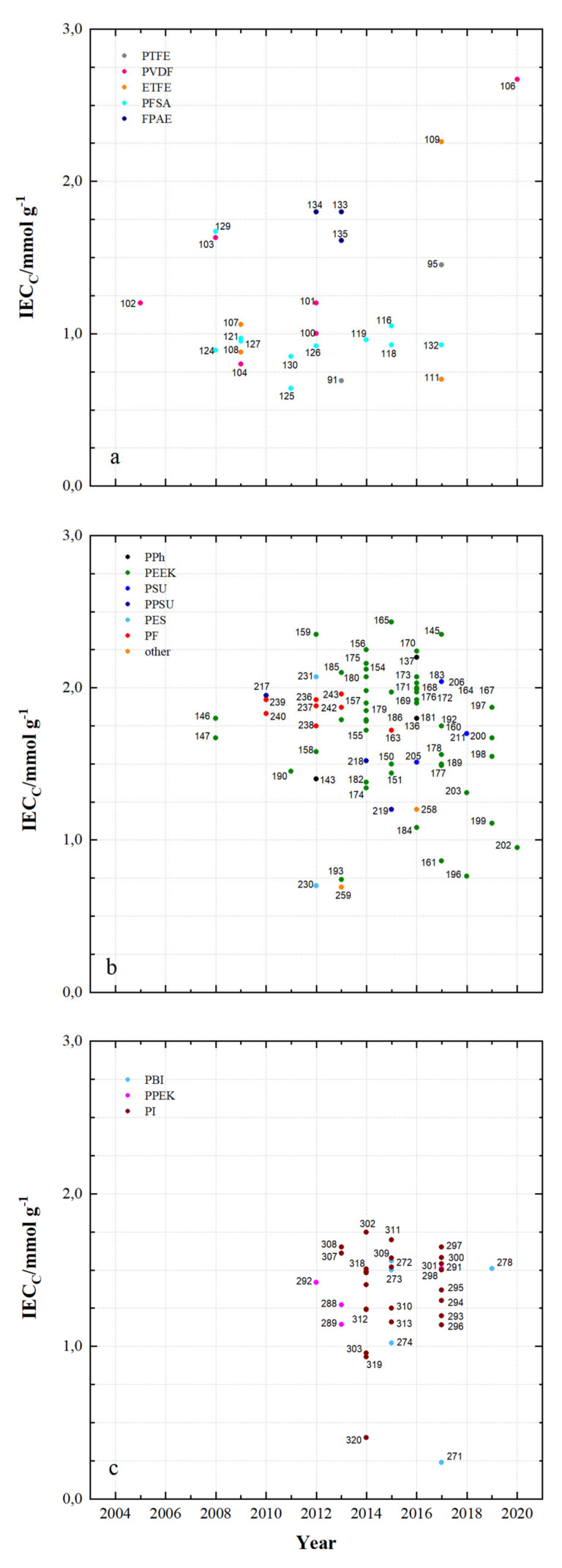
The ion-exchange capacity (IEC_c_) of tested polymer membranes in recent years: (**a**) fluoro-carbons, (**b**) hydro-carbons and (**c**) *N*-heterocycles.

For the DAPP membranes in Figure 7b the IEC_c_ is between 1.4 and 1.8 mmol g^−1^ and for the PEEK membranes between 0.74 (MS193) and 2.43 mmol g^−1^ (MS165). MS206 has an IEC_c_ of 2.04 mmol g^−1^, MS217 to MS219 an IEC_c_ of 1.2 to 1.95 mmol g^−1^, MS230 an IEC_c_ of 0.7 mmol g^−1^ and MS231 an IEC_c_ of 2.07 mmol g^−1^. The PF-based membranes have an IEC_c_ of 1.47 mmol g^−1^ to 1.96 mmol g^−1^. For MS259 and MS260 an IEC_c_ of 1.2 and 0.69 mmol g^−1^ was measured.

**Figure 8:**
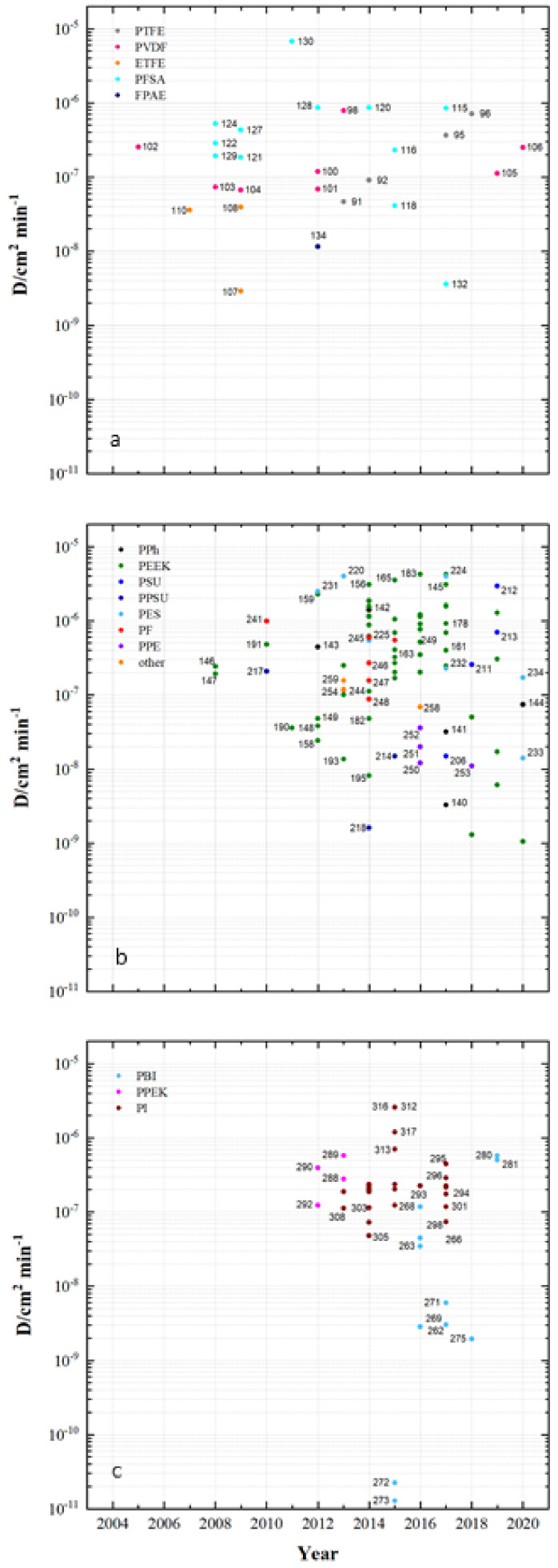
Measured diffusion coefficients of tested polymer membranes in recent years: (**a**) fluoro-carbons, (**b**) hydro-carbons and (**c**) *N*-heterocycles.

The PBI membranes in Figure 7c have an IEC_c_ of 0.24 to 1.56 mmol g^−1^, the PPEK membranes an IEC_c_ of 1.14 to 1.51 mmol g^−1^ and the PI membranes an IECc of 0.4 to 1.75 mmol g^−1^. In the development of membranes, IEC_c_ between 1 and 2 mmol g^−1^ is predominantly achieved. The PFSA membranes in the fluoro-carbon group increasingly exhibit IEC_c_ smaller than 1 mmol g^−1^. In the group of hydro-carbon-based membranes, especially when using PEEK, IEC_c_ of more than 2 mmol g^−1^ can be reached. Figure 8 shows the diffusion coefficients for vanadyl cations of membrane samples that have been tested and published for use in VRFB since 2005. Low diffusion coefficients lead to low vanadium cross-over during charging and discharging of the battery and therefore influences the coulombic efficiency. Diffusion coefficients (VO^2+^) from 2.9 × 10^−9^ to 6.72 × 10^−6^ cm^2^ min^−1^ for fluoro-carbon-based membranes and 1.6 × 10^−9^ to 4.2 × 10^−6^ cm^2^ min^−1^ for hydro-carbons, as well as 1.28 × 10^−11^ to 2.6 × 10^−6^ cm^2^ min^−1^ for *N*-heterocycles, have been published. Selected diffusion coefficients ranges:

- PTFE 4.62 × 10^−8^ to 7.1 × 10^−7^ cm^2^ min^−1^
- PVDF 6.7 × 10^−8^ to 7.9 × 10^−7^ cm^2^ min^−1^
- ETFE 2.9 × 10^−9^ to 3.9 × 10^−8^ cm^2^ min^−1^
- PFSA 3.6 × 10^−9^ to 6.72 × 10^−6^ cm^2^ min^−1^
- FPAE (MS134) 1.16 × 10^−8^ cm^2^ min^−1^
- PPh 3.3 × 10^−9^ to 1.4 × 10^−6^ cm^2^ min^−1^
- PEEK 1.05 × 10^−9^ to 4.2 × 10^−6^ cm^2^ min^−1^
- PSU 1.5 × 10^−8^ to 2.94 × 10^−6^ cm^2^ min^−1^
- PPSU 1.6 × 10^−9^ to 2.07 × 10^−7^ cm^2^ min^−1^
- PES 1.41 × 10^−8^ to 4 × 10^−6^ cm^2^ min^−1^
- PF 8.8 × 10^−8^ to 9.85 × 10^−7^ cm^2^ min^−1^
- PPE 1.1 × 10^−8^ to 3.6 × 10^−8^ cm^2^ min^−1^
- Other 6.9 × 10^−8^ to 1.56 × 10^−7^ cm^2^ min^−1^
- PBI 1.28 × 10^−11^ to 5.74 × 10^−7^ cm^2^ min^−1^
- PPEK 1.24 × 10^−7^ to 5.75 × 10^−7^ cm^2^ min^−1^
- PI 4.8 × 10^−8^ to 2.6 × 10^−6^ cm^2^ min^−1^.

The results on membrane thickness, ion-exchange capacity and vanadyl permeation summarized in Figure 6, Figure 7 and Figure 8, as well as the Supplementary data showing water uptake and anion-exchange capacity of membrane samples, are the most frequently investigated characteristics in publications on membrane development for VRFB cells. Other important properties are electrical resistance and ion-selectivity, for which the goal is to achieve a high proton conductivity, combined with the lowest vanadium-ion permeability possible. It is described in [113] that membrane thickness in particular has an influence on this and can be optimized accordingly.

With increasing ion-exchange capacity, water uptake increases in non-crosslinked membranes [84,114].

Due to the large number of data points, not every point in Figure 6, Figure 7 and Figure 8 is marked with the sample number from the following Table 5, Table 6, Table 7, Table 8, Table 9, Table 10, Table 11, Table 12, Table 13 and Table 14. The results pertaining water uptake, D_r_ as well as the ion-exchange capacity (IEC_A_) for AEM and AIEM are presented as Supplementary Materials.

The Table 5, Table 6, Table 7, Table 8, Table 9, Table 10, Table 11, Table 12, Table 13 and Table 14 list the membrane samples according to their membrane polymer structures, e.g., poly(sulfones). The tables start with the fluoro-carbon-based membranes followed by the hydro-carbon and the *N*-heterocycle-based membranes.

### 4.2. Membrane Impact on VRFB Cell Performance

**Figure 9:**
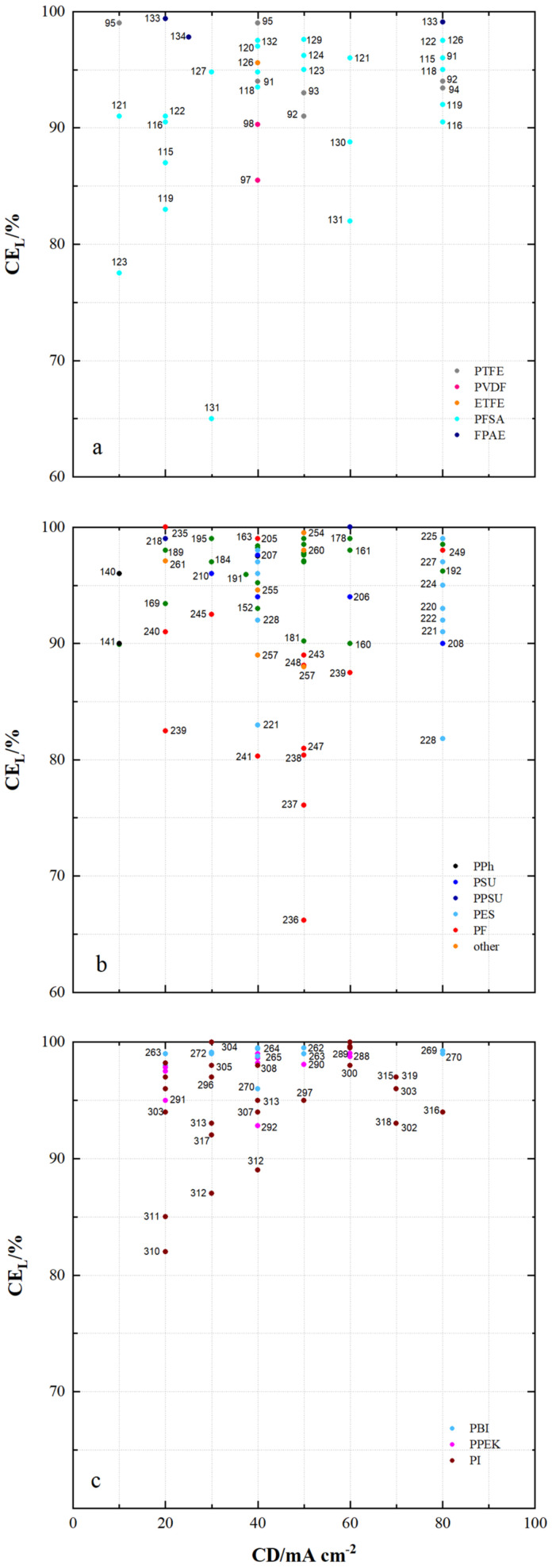
The energy efficiency of VRFB cells by using membrane samples at current densities < 100 mA cm^−2^: (**a**) fluoro-carbons, (**b**) hydro-carbons and (**c**) *N*-heterocycles.

**Figure 10:**
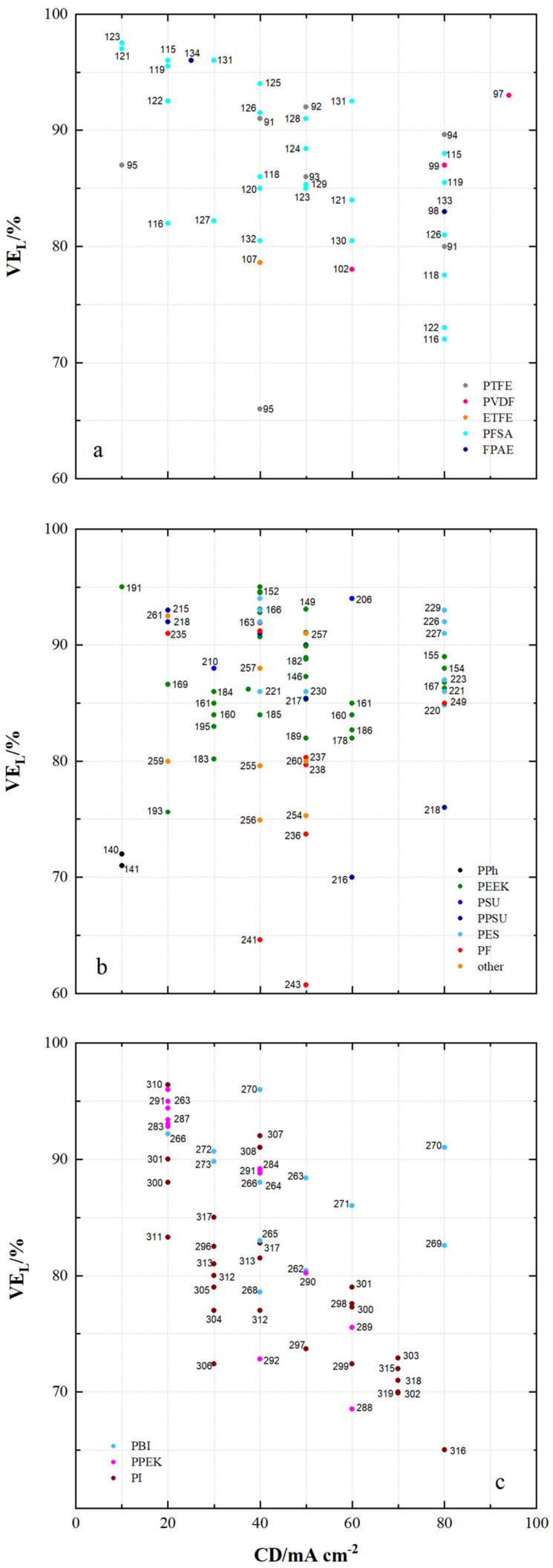
The voltage efficiency of VRFB cells by using membrane samples at current densities < 100 mA cm^−2^: (**a**) fluoro-carbons, (**b**) hydro-carbons and (**c**) *N*-heterocycles.

**Figure 11:**
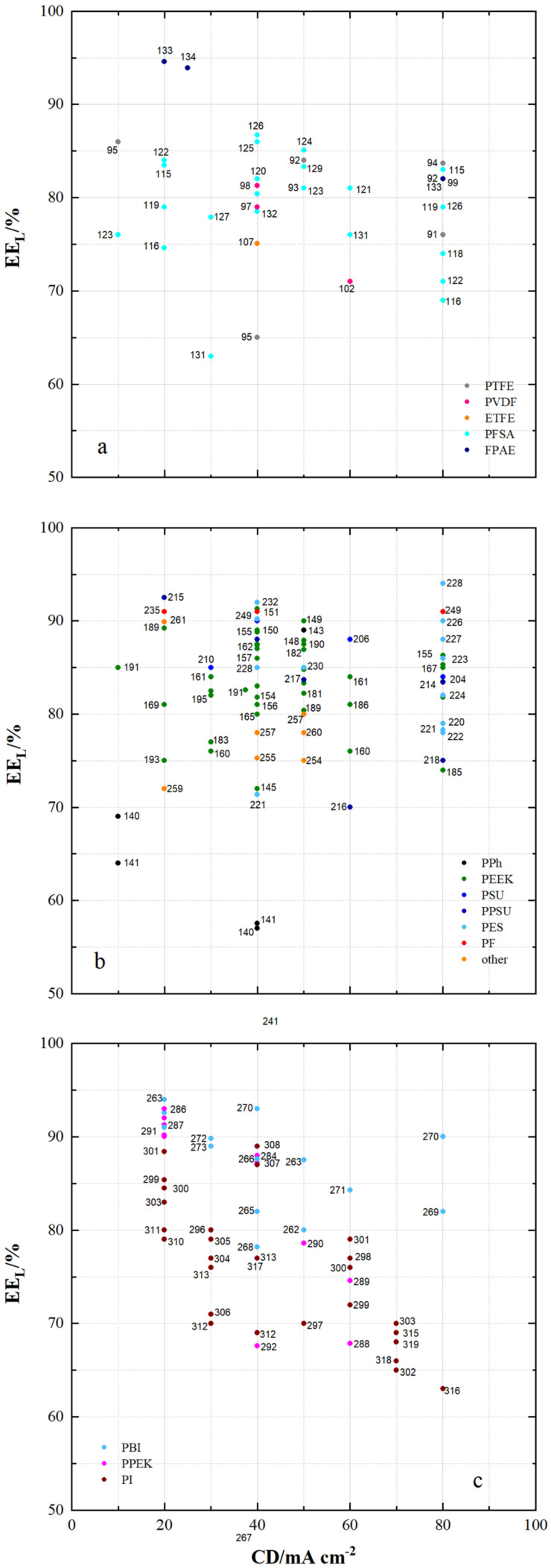
The energy efficiency of VRFB cells by using membrane samples at current densities < 100 mA cm^−2^: (**a**) fluoro-carbons, (**b**) hydro-carbons and (**c**) *N*-heterocycles.

**Figure 12:**
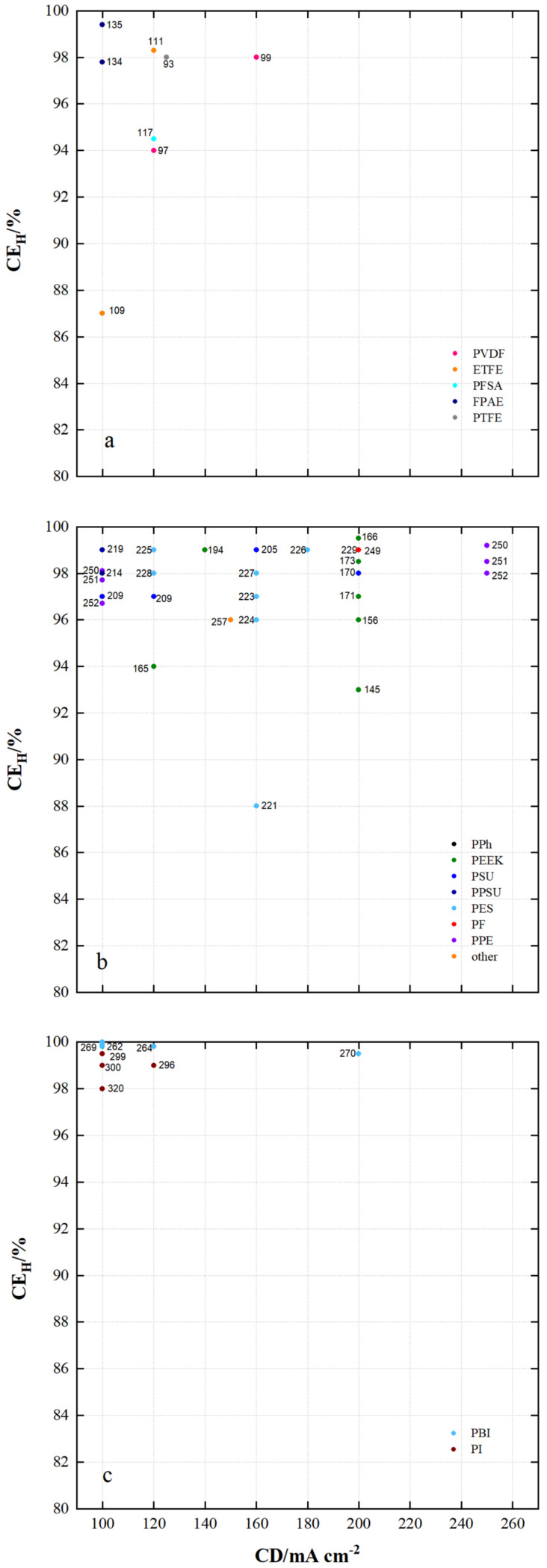
The coulombic efficiency of VRFB cells by using membrane samples at current densities ≥ 100 mA cm^−2^: (**a**) fluoro-carbons, (**b**) hydro-carbons and (**c**) *N*-heterocycles.

**Figure 13:**
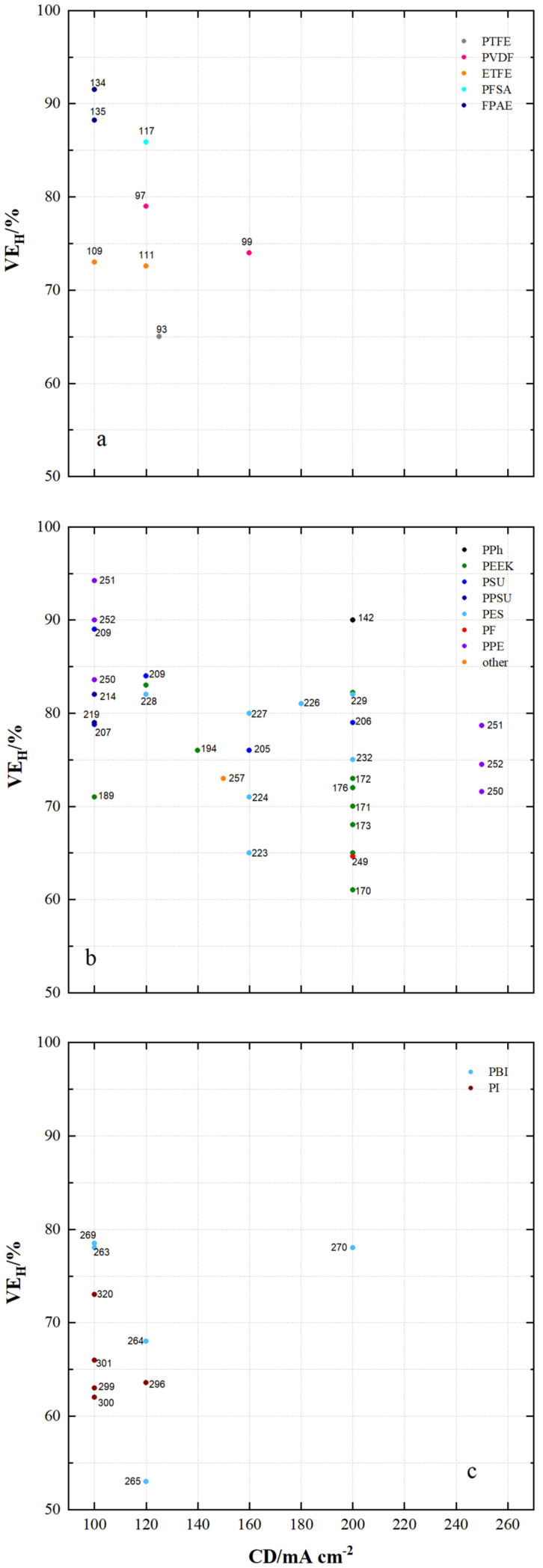
The voltage efficiency of VRFB cells by using membrane samples at current densities ≥ 100 mA cm^−2^: (**a**) fluoro-carbons, (**b**) hydro-carbons and (**c**) *N*-heterocycles.

**Figure 14:**
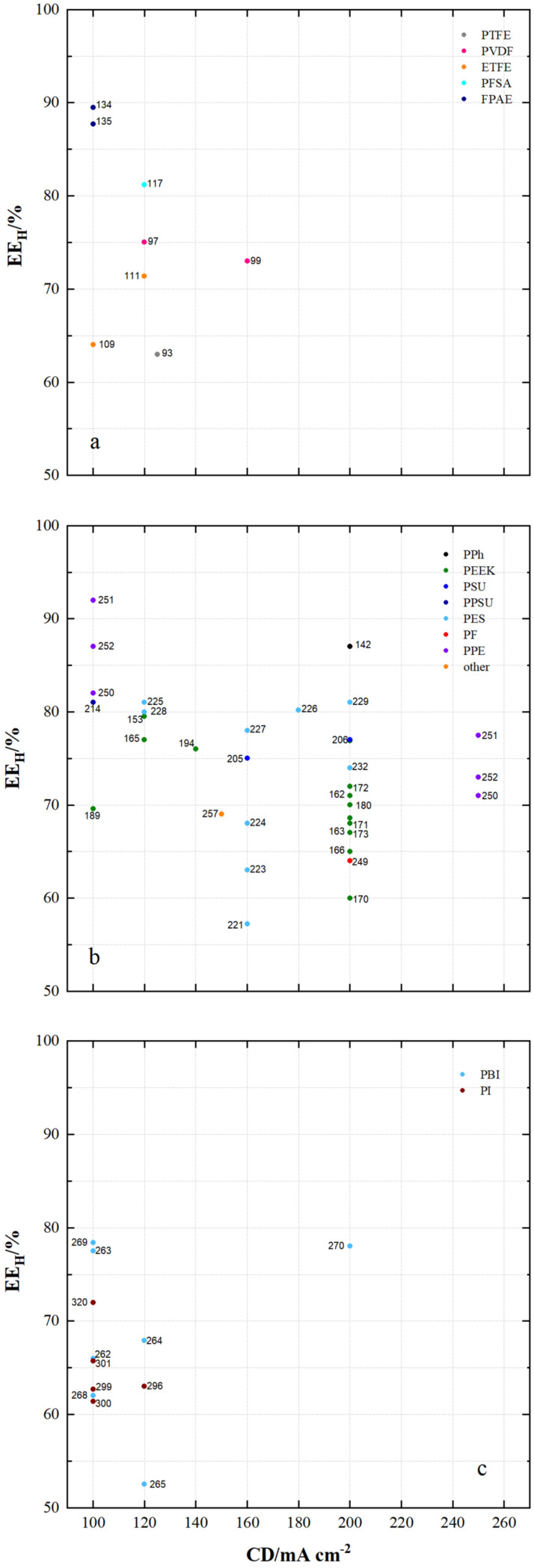
The energy efficiency of VRFB cells by using membrane samples at current densities ≥ 100 mA cm^−2^: (**a**) fluoro-carbons, (**b**) hydro-carbons and (**c**) *N*-heterocycles.

A VRFB cell is built up with frames, electrode felts, bipolar plates, electrolyte and membranes. Figure 9, Figure 10, Figure 11, Figure 12, Figure 13 and Figure 14 show the efficiencies of VRFB cells at different current densities. These cells are equipped with membranes from Table 5, Table 6, Table 7, Table 8, Table 9, Table 10, Table 11, Table 12, Table 13 and Table 14.

Figure 9 shows the coulombic efficiencies (CE_L_) of VRFB cells with current densities of less than 100 mA cm^−2^, constructed with either fluoro-carbon, hydro-carbon or *N*-heterocycle-based samples.

CE_L_ ranging from 65% to 99% were achieved with fluoro-carbon-based membrane samples, CE_L_ from 66% to 100% with hydro-carbon-based membranes and CE_L_ from 82% to 100% with *N*-heterocycle-based membranes.

In fluoro-carbons (Figure 9a), VRFB cells using MS95 (PTFE, dhe, CEM), MS133 (FPAE, dho, CEM), MS134 (FPAE, dho, CEM), MS132 (PFSA, dhe, CEM), MS129 (PFSA, dhe, CEM), MS126 (PFSA, dhe, CEM), MS122 (PFSA, dhe, AIEM) and MS107 (ETFE, dho, AIEM) achieve high CE_L_ of at least 98%.

In hydro-carbon-based membranes VRFB cells with modified PEEK membranes MS195 (PEEK, dho, AEM), MS161 (PEEK, dhe, CEM) and MS178 (PEEK, dhe, CEM) also achieve high CE_L_ of at least 98%. Furthermore, MS235 (PF, dho, AEM), MS254 (other, dho, AEM), MS195 (PSU, dho, AEM), MS218 (PPSU, dho, CEM), MS205 (PSU, sym, AEM) and MS225 (PES, asym, CEM) achieve similarly high CE_L_. In Figure 9c VRFB cells with PBI membranes MS263 (PBI, dho, AEM*), MS264 (PBI, dho, AEM*), MS262 (PBI, dho, AEM*), MS272 (PBI, dho, AEM*), MS269 (PBI, asym, AEM*) and MS270 (PBI, sym, AEM*) show CE_L_ of at least 98% as well. The VRFB cells with PPEK-based membranes (MS282-MS291) all show a CE_L_ of at least 98% except for MS292 (PPEK, dho, CEM).

VRFB cells equipped with PI membranes MS298 (PI, dho, CEM), MS301 (PI, dho, CEM), MS304 (PI, dho, AIEM*), MS305 (PI, dho, CEM) and MS308 (PI, dhe, AIEM*) all reach high CE_L_ of at least 98%.

In some cases, the CE_L_ depend on the membrane thickness. This is observed with the PTFE-based membranes (Figure 9a). The CE_L_ ranges from 94% (MS92/25 µm) to 99% (MS96/70 µm). The VRFB equipped with MS91 (45 µm) shows a CE_L_ of 96%. Other examples do not show this correlation, e.g., MS111 with a thickness of 25 µm and a CE_L_ of 98% at 120 mA cm^2^. In summary, it appears to be possible to reach high CE_L_ with membrane polymers from all three groups.

Figure 10 shows the voltage efficiencies (VE_L_) of VRFB cells using the specified membrane samples with current densities less than 100 mA cm^−2^.

The VE_L_ are 66% to 97.5% with fluoro-carbon-based membranes, 60.7% to 95% with hydro-carbon-based membranes and 65% to 96.4% with *N*-heterocycle-based membranes.

For the fluoro-carbon-based membranes in Figure 10a, at a current density of 80 mA cm^−2^, the highest VE_L_ of VRFB cells are achieved using MS94 (PTFE, dhe, CEM), MS115 (PFSA, dhe, AIEM*), MS99 (PVDF, asym), MS119 (PFSA, dhe, CEM), MS98 (PVDF, asym), MS126 (PFSA, dhe, CEM) and MS91 (PTFE, dhe, CEM). The minimum VE_L_ achieved here is 80%.

For the hydro-carbon-based membranes in Figure 10b, at a current density of 80 mA cm^−2^, the highest VE_L_ of VRFB cells are obtained using MS229 (PES, asym, CEM), MS226 (PES, sym, AIEM), MS227 (PES, asym, AIEM), MS155 (PEEK, dho, CEM), MS154 (PEEK, dho, CEM), MS174 (PEEK, dhe, CEM), MS192 (PEEK, dho, CEM), MS167 (PEEK, dhe, CEM), MS176 (PEEK, dhe, CEM), MS214 (PPSU, dho, AEM), MS208 (PSU, asym, AIEM), MS194 (PEEK, dho, AEM) and MS249 (PF, dho, CEM). The minimum VE_L_ achieved here is 85%.

For the *N*-heterocycle-based membranes in Figure 10c, at a current density of 80 mA cm^−2^, the highest VE_L_ of VRFB cells are achieved using MS270 (PBI, sym, AEM*) and MS269 (PBI, asym, AEM*). All VE_L_ measured here are above 80%.

VE_L_ of at least 95% are reached by VRFB cells at lower current densities with MS123 (PFSA, dhe, AIEM), MS121 (PFSA, dhe, CEM), MS115 (PFSA, dhe, AIEM*), MS119 (PFSA, dhe, CEM),

MS134 (FPAE, dho, CEM), MS131 (PFSA, dhe, AIEM*), MS191 (PEEK, dhe, CEM), MS152 (PEEK, dho, CEM), MS310 (PI, dhe, CEM), MS270 (PBI, sym, AEM*), MS263 (PBI, dho, AEM*) and MS291 (PPEK, dho, CEM).

Figure 10a,c show the tendency of decreasing VE_L_ with increasing current density, which cannot be observed as a trend for hydro-carbon-based membranes. This can be explained by a lower electrical resistance, which can be achieved with aromatic polymers with high degrees of sulfonation.

Figure 11 shows the energy efficiency (EE_L_) of VRFB cells using the specified membrane samples with current densities less than 100 mA cm^−2^.

For VRFB cells with fluoro-carbon-based membrane samples, the EE_L_ ranges from 63% to 95%. For hydro-carbon-based membranes the EE_L_ ranges from 57% to 94% and for N-heterocycle-based membranes the EE_L_ ranges from 63% to 94%.

For VRFB cells with fluoro-carbon-based membranes, high EE_L_ of at least 85% are measured with MS133 (FPAE, dho, CEM), MS134 (FPAE, dho, CEM), MS126 (PFSA, dhe, CEM), MS125 (PFSA, dhe, CEM), MS95 (PTFE, dhe, CEM) and MS124 (PFSA, dhe, AIEM*).

For VRFB cells with hydro-carbon-based membranes, EE_L_ of at least 90% are achieved with MS215 (PPSU, dho, AEM), MS235 (PF, dho, AEM), MS232 (FPAE, dhe, CEM), MS151 (PEEK, dhe, CEM), MS249 (PF, dho, CEM), MS149 (PEEK, dho, CEM) and MS228 (PES, dhe, CEM). With many other membranes from Figure 11b, EE_L_ of at least 85% were achieved.

In VRFB cells with *N*-heterocycle-based membranes, high EE_L_ of at least 85% are measured with MS263 (PBI, dho, AEM*), MS286 (PPEK, dho, AEM), MS287 (PPEK, dho, AEM), MS291 (PPEK, dho, CEM), MS301 (PI, dho, CEM), MS299 (PI, dho, CEM), MS272 (PBI, dho, AIEM*), MS273 (PBI, dho, AIEM*), MS308 (PI, dhe, AIEM*), MS284 (PPEK, dho, AEM), MS266 (PBI, sym, AEM*), MS307 (PI, dho, CEM), MS263 (PBI, dho, AEM*) and MS270 (PBI, sym, AEM*).

The improvement of the EE_L_ e.g., at 80 mA cm^−2^ is caused by the optimization of different membrane properties. The series of SFPAE membranes (28, 45, 80 µm) in [114] showed a different energy efficiency due to different membrane thickness. A water uptake of 30% leads to the highest energy efficiency of 90% at 50 mA cm^−2^ by using PEEK-based CEM.

It has been shown that with a range of different membrane samples, high EE_L_ of over 90% at current densities of less than 100 mA cm^−2^ are feasible. These high efficiencies are achieved by cells with dense fluoro-carbon and dense hydro-carbon-based membranes as well as dense and symmetrically porous *N*-heterocycle-based membranes. The respective membranes are composed of different polymers and can be assigned to CEM, AEM, AEM* and AIEM*.

Figure 12 shows the coulomb efficiencies (CE_H_) of VRFB cells with current densities of at least 100 mA cm^−2^ using the specified membrane samples.

In the VRFB cells from Figure 12a equipped with the fluoro-carbon-based membrane samples, CE_H_ of 87% to 99.5% were measured. The VRFB cells equipped with the hydro-carbon-based membranes shown in Figure 12b achieved CE_H_ from 88% to 99.5%. Using the *N*-heterocycle-based membranes shown in Figure 12c, CE_H_ of 98% to 99.8% were realized.

In fluoro-carbon-based membranes VRFB cells with MS135 (FPAE, dhe, CEM), MS111 (ETFE, dho, AIEM), MS93 (PTFE, sym, -) and MS97 (PVDF, sym, -) also achieve high CE_H_ of at least 98%.

In hydro-carbon-based membranes VRFB cells with MS219 (PPSU, dhe, AIEM*), MS214 (PPSU, dho, AEM), MS250 (PPE, dho, AEM), MS225 (PES, asym, CEM), MS228 (PES, dhe, CEM), MS194 (PEEK, dho, AEM), MS227 (PES, asym, AIEM*), MS205 (PSU, sym, AEM), MS226 (PES, sym, AIEM), MS166 (PEEK, dho, CEM), MS229 (PES, asym, CEM), MS249 (PF, dho, CEM), MS173 (PEEK, dhe, CEM), MS170 (PEEK, dho, CEM), MS251 (PPE, dho, AEM) and MS252 (PPE, dho, AEM) reach high CE_H_ of at least 98%.

For the *N*-heterocycle-based membranes all VRFB cells shown reach CE_H_ of at least 98%. These include MS269 (PBI, asym, AEM*), MS262 (PBI, dho, AEM*), MS264 (PBI, dho, AEM*), MS299 (PI, dho, CEM), MS300 (PI, dho, CEM), MS320 (PI, dho, CEM), MS296 (PI, dho, CEM) and MS270 (PBI, sym, AEM*).

Low CE_H_ were measured with MS109 (ETFE, dho, CEM), MS145 (PEEK, dho, CEM) and MS221 (PES, sym, CEM). It can be assumed that the water uptake of 181% for MS109 and 88% for MS145 as well as large pores for MS221 during cycling led to excessive electrolyte transfer and thus to charge loss.

Lower D_c_ measured for the membranes in Figure 12 enable higher coulombic efficiencies. For example, in contrast to MS145 (D_c_ = 3.06 × 10^−6^/CE_H_ = 93%) higher CE_H_ are realized with MS162 (D_c_ = 1.04 × 10^−7^/CE_H_ = 97%), MS163 (D_c_ = 1.67 × 10^−7^/CE_H_ = 99%) and MS173 (D_c_ = 3.5 × 10^−7^/CE_H_ = 99%) at the same current density of 200 mA cm^2^.

Figure 13 shows the voltage efficiencies (VE_H_) of VRFB cells with current densities of at least 100 mA cm^−2^ using the specified membrane samples. The VE_H_ of VRFB cells using the fluorocarbon-based membrane samples shown in Figure 13a ranges from 65% to 92%.

VE_H_ of 61% to 99% are achieved using the hydro-carbon-based membranes shown in Figure 12b and VE_H_ of 53% to 78% using the *N*-heterocycle-based membrane samples shown in Figure 12c.

High VE_H_ of at least 80% are measured with MS134 (FPAE, dho, CEM), MS135 (FPAE, dhe, CEM), MS117 (PFSA, dhe, CEM), MS251 (PPE, dho, AEM), MS252 (PPE, dho, AEM), MS209 (PSU, sym, AEM), MS142 (DAPP, dho, AEM), MS250 (PPE, dho, AEM), MS214 (PPSU, dho, AEM), MS209 (PSU, sym, AEM), MS228 (PES, dhe, CEM), MS164 (PEEK, dho, CEM), MS225 (PES, asym, CEM), MS226 (PES, sym, AIEM), MS229 (PES, asym, CEM) and MS145 (PEEK, dho, CEM).

For VRFBs equipped with *N*-heterocycle-based membranes, the highest VE_H_ with MS269 (PBI, asym, AEM*) is 78.5%, with MS263 (PBI, dho, AEM*) and with MS270 (PBI, sym, AEM*) 78%.

With the exception of MS270 in Figure 13c, the results in Figure 13a–c show the tendency of the VE_H_ to decrease with increasing current density.

Figure 14 shows the energy efficiency (EE_H_) of VRFB cells with a current density of at least 100 mA cm^−2^ using the specified membrane sample.

Energy efficiencies of 63% to 89.5% were achieved with fluoro-carbon-based membranes, 57.2% to 92% with hydro-carbon-based membranes and 52.5% to 78.4% with *N*-heterocycle-based membranes.

Using fluoro-carbon-based membranes, VRFB cells with MS134 (FPAE, dho, CEM) achieve an EE_H_ of 89.5% at a current density of 100 mA cm^−2^, with MS135 (FPAE, dhe, CEM) an EE_H_ of 87.7% at a current density of 100 mA cm^−2^ and with MS117 (PFSA, dhe, CEM) an EE_H_ of 81% at a current density of 120 mA cm^−2^ at a current density of 100 mA cm^−2^.

Using hydro-carbon-based membranes, VRFB cells can be obtained with MS251 (PPE, dho, AEM), MS252 (PPE, dho, AEM), MS250 (PPE, dho, AEM), MS214 (PPSU, dho, AEM), MS225 (PES, asym, CEM), MS228 (PES, dhe, CEM), MS226 (PES, sym, AIEM), MS229 (PES, asym, CEM) and MS142 (DAPP, dho, AEM) and have an EE_H_ of at least 80%.

In VRFB cells with N-heterocycle-based membranes, the highest EE_H_ are between 70% and 80%. MS269 (PBI, asym, AEM*), MS263 (PBI, dho, AEM*), MS270 (PBI, sym, AEM*) and MS320 (PI, dho, CEM) were used.

**Figure 15:**
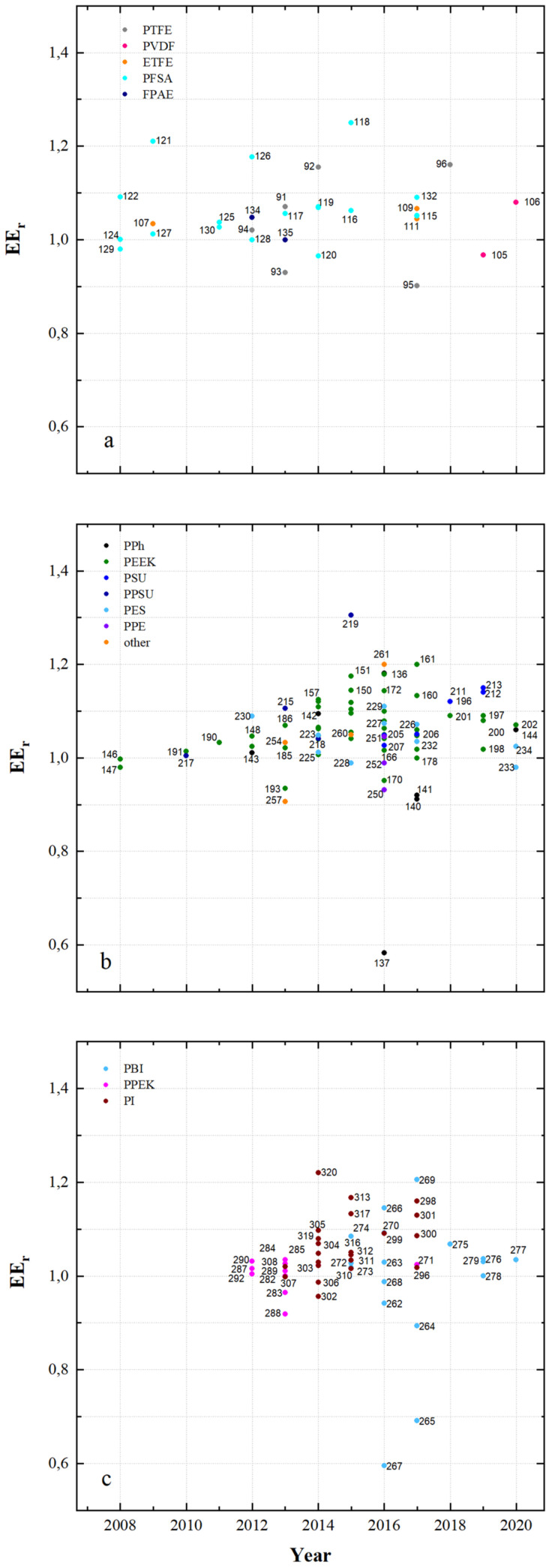
Energy efficiency ratios in recent years: (**a**) fluoro-carbons, (**b**) hydro-carbons and (**c**) *N*-heterocycles.

Figure 15 shows the EE_r_ (calculated with the reference membrane from Table 5, Table 6, Table 7, Table 8, Table 9, Table 10, Table 11, Table 12, Table 13 and Table 14) of VRFB cells and the respective publication year.

An EE_r_ of 0.901 to 1.250 is obtained with fluoro-carbon-based membranes, an EE_r_ of 0.907 to 1.305 is obtained with hydro-carbon-based membranes and an EE_r_ of 0.919 to 1.220 is obtained with *N*-heterocycle-based membranes.

With MS118 (PFSA, dhe, CEM), MS121 (PFSA, dhe, CEM), MS126 (PFSA, dhe, CEM) and MS92 (PTFE, dhe, CEM), an EE_r_ of at least 1.1 was achieved for the fluorocarbon-based membranes. The hydro-carbon-based membranes were tested with MS219 (PPSU, dho, AIEM*), MS161 (PEEK, dhe, CEM), MS136 (DAPP, dho, CEM), MS151 (PEEK, dhe, CEM), MS150 (PEEK, dho, CEM), MS172 (PEEK, dhe, CEM), MS160 (PEEK, dho, CEM) and MS157 (PEEK, dho, CEM) and achieved a high EE_r_ of at least 1,1. Among others, MS320 (PI, dho, CEM), MS269 (PBI, asym, AEM*), MS313 (PI, dhe, AIEM*), MS266 (PBI, sym, AEM*), MS298 (PI, dho, CEM), MS301 (PI, dho, CEM) and MS317 (PI, dhe, AIEM*) achieved a high EE_r_ of at least 1.1.

Potential for improvement of the VRFB can be seen especially in the hydro-carbon and *N*-heterocycle-based membranes in Figure 15b,c. This improvement represented by EE_r_ is also observed with the fluoro-carbons, however, in weaker expression.

In conclusion, Figure 15 and Table 15 both show that membrane change often leads to improved energy efficiency under otherwise identical test conditions.

Publications show, in part, the influence of the polymer and membrane properties on VRFB cell performance. Water uptake and the degree of functionalization can be optimized by the use of cross-linkers [35]. An optimum of 18% (WU) is determined for the PPE-based membranes (dho, AEM) [35]. At this optimum and a current density of 100 mA cm^−2^ the maximum CE, VE and EE is 97.7%, 94% and 92%. Here, an EE_r_ of 1.045 compared to N212 can be achieved. Permeability can be improved by introducing positive charges into the polymer [162]. This leads to the ability of the membrane to keep CE high and self-discharge of a VRFB cell low.

An important property of polymer membranes is the ion-selectivity which can be determined by proton conductivity and permeation experiments. This selectivity can further be optimized by adjusting the thickness of CEM [113] to maximize the CE, VE and EE of the VRFB cell.

Ionically cross-linked blend membranes [34] represent one of the well-balanced compromises regarding these properties. The ionic cross-linking of PBI and sulfonated PPSU enables reduced water uptake combined with comparatively high IEC_c_ and high proton conductivity. This type of membrane with a thickness of 50 µm enables a high EE of 77% and an EE_r_ of 1305 compared to N117 at 100 mA cm^−2^.

Good results can also be achieved with PBI membranes containing enhanced targeted porous structures. Using symmetric porous structures, PBI membranes from [193] and [195] enable energy efficiencies of 87% and 90%. The asymmetrically porous PBI membrane from [194] enables a comparatively high energy efficiency of 82% as well.

Furthermore, it is possible to design porous membranes with neutral polymers such as PVDF (MS99, asym) or PTFE (MS93, sym). These appear to have improved long-term stability [91,118].

While Table 5, Table 6, Table 7, Table 8, Table 9, Table 10, Table 11, Table 12, Table 13 and Table 14 provide an exhaustive list of EE_r_ values, Table 15, for the sake of brevity, contains only the fifteen flow-battery targeted membrane samples which showed the highest values during our investigation.

## 5. Cycle Stability

Various methods are used to evaluate the stability of membranes. It is possible to determine the weight loss over time at a certain temperature by way of mass balance. This is done using swelling tests in aggressive media such as Fenton’s reagent or charged VRFB catholytes. Another method is VRFB cycling tests, which plot the achieved battery performance over a number of cycles graphically. This test is performed to evaluate the stability of the membrane for a given number of cycles [144] or to determine the time of failure of the membrane [206].

Table 16 shows results of VRFB’s cycling tests. In most cases, a current density of 40–80 mA cm^−2^ was used to show cycle stability. Cycle stability at current densities of 120–200 mA cm^−2^ was demonstrated in some cases [35,45,74,103,134,199]. Furthermore, the electrolyte quantities in these cycling tests varied. A comparatively high cycle stability of 13000 cycles was demonstrated with a symmetrically porous PBI membrane [195]. A total of 6000 cycles were achieved with a symmetrically porous PSU membrane (AEM, cross-linked) [102]. 4000 charge/discharge cycles were performed with a partially fluorinated and vinylimidazole-based AEM [41]. 1000 cycles were completed with an asymmetrically porous PVDF membrane [118] and a sPEEK-based cation exchange membrane [144]. Many other results with cycles between 50 and 13,000 can be found in Table 16.

## 6. Membrane Costs

The cost of a VRFB varies with its electrical power (stack size) and available storage capacity (volume of battery electrolyte). The cost proportion of the installed components can therefore vary greatly. A cost analysis conducted by the U.S. Department of Energy (DOE) showed that the cost proportion of the membrane, measured against the total system, is 44% for a plant with a storage capacity of 0.25 MWh and 27% for a plant with a storage capacity of 4 MWh [35]. The cost proportion of the system is stated to be even lower at 10–15% in [65]. In relation to the stack costs, however, a cost proportion of about 40% has been assumed for the use of Nafion [52,65,86,92,185,193,197], whereby also cost ranges of 30–50% of the stack were mentioned [144]. The high specific cost of Nafion (500–800 USD m^−2^) [86,189] is reported disparately, since with decreasing mass per square meter and different membrane thickness (50.8 µm to 183 µm) as well as different purchase quantities, the price varies. In [46] is mentioned that a quantity of 0.3 × 20 m^2^ N212 is about 50% cheaper than N115. In [163] N212 is quoted at 225 USD m^-^^2^. Referring to [46] this results in a cost saving of 12% to 25% if N212 is preferred to a N115 membrane. Substituting the N115 membrane for a Vanadion membrane reduces the cost from 331 USD kWh^−1^ to 251 USD kWh^−1^ for a 1 MWh plant [27]. Furthermore, rough cost estimates are given for published flow-battery targeted membranes. It is assumed that a PES-based membrane is about 1/10 of the price of N115 [174]. PEEK are generally said to have lower production costs due to the aromatic main chain [33]. For PPSU-based membranes, the price could be about 1/400 of Nafion [48]. For partially fluorinated sulfonated PI membranes the manufacturing cost is 167 USD m^−^^2^ [206]. With material costs of 100 USD m^−^^2^, the cost of PAEK membranes appears to be lower than the cost of Nafion [152]. The cost for PSU membranes is 21 to 24 USD m^−^^2^ [163]. Further, when using PSU membranes, a cost saving of 1/20 compared to Nafion is reported [165]. Table 17 gives an overview of low-cost flow-battery targeted membranes in recent years.

C. Minke et al. have dealt with the costs of VRFB, in particular the costs of membranes more extensively. This is how a cost proportion of 37%, for a 250 kW stack using Nafion membrane, is calculated [214]. The use of sPEEK membranes could reduce the cost proportion to 8%. This would reduce the cost of a 250 kW stack from 219,000 EUR to 150,000 EUR. They also describe that the specific price of membranes depends on the production volume. A calculation in [215] shows that the price of Nafion can be reduced from 300 USD m^−^^2^ to approximately 20 USD m^−^^2^ if the production quantity is increased from 0.01 to 10 million square meters per year. The comprehensive listing of membrane costs in [10] describes a current cost range of 16–451 EUR m^−^^2^.

**Figure 16:**
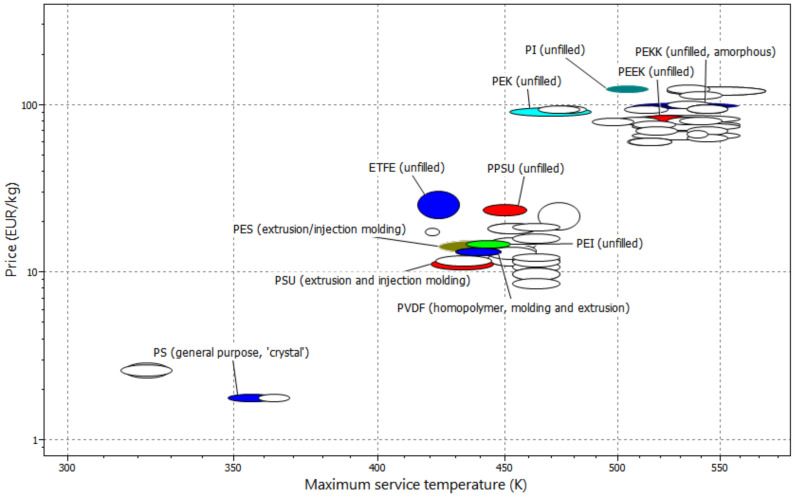
Commercial polymer products: specific costs and maximum service temperature [216].

Looking at the raw material prices in the plastics industry, differences can be seen in the specific costs for polymer granulates, which are used in various flow-battery targeted membranes. The Cambridge Engineering Selector database [216] provides an overview (Figure 16). PS and PP are traded at significantly less than 5 EUR kg^−1^. PSU, PES, ETFE, PVDF, PTFE and PPSU are traded in the range from 10 to 12 EUR kg^−1^. The high-performance polymers PEEK, PEK, PEKK and PI range from 70 to 110 EUR kg^−1^.

## 7. Conclusions

By now, numerous flow-battery targeted membranes for the VRFB exist. Most developments show improved VRFB performance when compared to VRFB equipped with reference (Nafion) membranes.

Less often, the stability of membranes is investigated at high cycles of significantly more than 1000. From a technical point of view, information on this has a similar significance as the demonstration of VRFB performance with a new membrane.

When stating costs, only rough estimates can be made usually. The cost of up scaling, e.g., choice and planning of production technology, are often not taken into account. Fundamental examples of the influence of membrane properties on VRFB performance were described in Section 4.2. They can be considered as an approach for the development of membranes based on other, cheaper or chemically more stable polymers.

Results from PFSA membrane modifications lead to the conclusion of improved VRFB performance. These modifications, however, may lead to increased production costs through potential additional steps during manufacturing. PFSA membranes are known for their chemical stability, which was also demonstrated in cycling tests with new “low-cost” or fluorine-free membranes. 1000 and more charge and discharge cycles were achieved with MS98 (PVDF, asym, -), MS167 (PEEK, dhe, CEM), MS205 (PSU, sym, AEM), MS254 (QPTM, dho, AEM) and MS270 (PBI, sym, AEM*). A lot of membrane modifications can be found in hydro-carbon-based membranes, where DAPP is investigated in addition to commercially available PEEK or PSU.

Which part of a performance improvement can be attributed to a specific membrane property can generally not be formulated in concrete figures, as these partly influence each other. For example, changes in ion-exchange capacity lead to changes in swelling properties, which affect thickness, water uptake and ultimately selectivity.

For cost optimized VRFB manufacturing, membrane production must be a continuous process on an industrial scale. In addition to investment and operating costs, raw material prices are a large influence for the specific costs of membranes produced in a large scale. In order to determine the material costs, it is therefore necessary to know the material composition of the membrane and the amount of operating materials required for all production steps.

On the basis of the data from this study, we conclude that some suggestions with reference to membrane type for different operating modes of the VRFB can be made. Dense AEM and N-heterocycle-based membranes, especially PBI membranes, are suitable for lowest discharge of the VRFB. Symmetric and asymmetric porous membranes as well as CEM enable VRFB operation at high current densities. AIEM and dense heterogeneous CEM are the choice for operation mode with highest energy efficiency (Table 18). The cost column in Figure 16 shows the specific cost range for the three material groups fluorocarbon, hydro-carbon and *N*-heterocycle-based membranes. Of course, PVDF and ETFE-based materials are in the cost range of PSU or PES (Figure 16), but the expansive PFSA materials increase the average value. The manufacturing of dense polymer films is generally easier and cheaper than making membranes with a special porosity.

VRFB performance, high chemical stability and reduced costs will continue to play an important role in future membrane research. For promising membrane developments, long-term cycling experiments are recommended, whereby the membrane is examined before and after with regard to its chemical and structural change.

There is still research potential in the choice of materials for membrane development using polymer products with a price well below 10 EUR kg^−1^. For lithium-ion batteries a list of different coated porous polyolefin separators was published in 2016 [217]. The Poly(ethylene) (PE), poly(propylene) (PP) and PE/PP-based low-cost separators can be a good starting material for making VRFB membranes, too. In 2020 such a kind of VRFB membrane was made by coating a hydrophilic poly(ethylene) separator with PBI [198].

The influence of the membrane composition regarding proton conductivity and vanadyl permeation is relatively well known, but the influence of the membrane structure is mostly unknown. Future research and development approaches could include the in-depth investigation of membrane structures and their influence on VRFB performance. This is seldom considered regarding ion-exchange membranes, even though dense CEM, AEM or AIEM also have electrolyte-filled pores and channels.

The use of commercial polymers is just as advantageous as the use of polymerizable monomers. If large production quantities are considered, the question also arises as to what a suitable recycling concept for discarded membranes could look like. If these membranes were to be selected for thermal recycling, fluorine-free materials would lower cost. This should also be taken into account for other membrane additives.

Future efforts to enhance the design of membranes for VRFB could still be the development of new polymer materials as well as manufacturing technology innovations. Generally, some “simple” and fundamental facts should be taken into account, when designing membranes for VRFB-based on polymers:

- Dimensional stability after soaking the dry membrane in battery electrolyte or water is very important to keep the ion channels diameters as small as possible.
- For sulfonated polymers as a proton conductor in the membranes, it should be taken into account that its acidity is dependent on the polymer used and influences the proton conductivity.
- The thickness of the membrane (length of ion channels) should be optimized for high selectivity.
- As many ion channels as possible should be aimed for good conduction between the two half-cells.

The polymer chemistry of a membrane and its interaction with the battery electrolyte not only, but also the membrane morphology allow special membranes for enhanced VRFB performance in low self-discharge, high current density or high energy efficiency mode. The degree of sulfonation and covalent or ionic cross-linking of polymers are important methods to enhance the membrane morphology. This was shown with some membranes mentioned in this study. Polymer cross-linking should be focused when designing membranes with high degree of sulfonation. Additionally, self-ordering polymers, like copolymers or polymers with crystalline proportions could be an option to control membrane morphologies on a molecular scale or to enhance its chemical stability.

Manufacturing technology could include dielectrophoresis units to enhance the design of membranes, too. Dielectrophoresis is a method to separate materials with different dielectric properties. Due to the fact that composite membranes, containing a proton conductor and, e.g., a hydrophobic matrix, consist of materials with different dielectric constants it is possible to align the proton conductor as ion channels between the two surfaces of a flat sheet membrane in an electric field during manufacturing. This might influence proton conductivity and H^+^/V selectivity.

Furthermore, it might be possible to increase the resistance to the highly oxidizing electrolyte of the positive half-cell by additional coating strategies.

## Figures and Tables

**Table 1:** Overview of review papers considering vanadium redox flow battery membranes.

Year	Journal	Title	Main Focus	Ref.
2011	Energy Environ. Sci.	Ion exchange membranes for vanadium redox flow battery (VRB) application	all aspects related to IEMs that are of relevance to understand IEMs for VRFB	[11]
2011	ChemSusChem	Membrane Development for Vanadium Redox Flow Batteries	basic scientific issues associated with membrane use in VRFBs	[12]
2012	Membranes	Membranes for VRFB Applications	membranes for all-vanadium redox flow battery which has received the most attention.	[13]
2013	Electrochimica Acta	Review of material research and development for vanadium redox flow battery applications	a historical overview of materials research and development	[15]
2014	Energy Environ. Sci.	Anion-exchange membranes in electrochemical energy systems	technological and scientific limitations and the future challenges related to the use of anion-exchange membranes	[16]
2015	J.o.Nanomaterials	Recent development of Nanocomposite Membranes for Vanadium redox Flow Batteries	efforts in developing nanocomposite membranes	[14]
2015	RSC Adv.	Recent development of polymer membranes as separators for all-vanadium redox flow batteries	new cation exchange membranes, anion exchange membranes, amphoteric ionexchange membranes, and non-ionic porous materials	[17]
2015	RSC Adv.	A review on recent developments of anion exchange membranes for fuel cells and redox flow batteries	developments in the synthesis and applications of AEMs in the field of electrochemical energy conversion and storage	[18]
2017	Chem. Soc. Rev.	Porous membranes in secondary battery technologies	understanding of the preparation–structure– performance relationship	[19]
2017	Journal of Membrane Science	Ion exchange membranes: New developments and applications	new iem materials	[20]
2018	Chem. Commun.	Ion conducting membranes for aqueous flow battery systems	porous membranes, different flow batteries	[21]
2018	Energy Environ. Sci.	Review of electrical energy storage technologies, materials and systems: challenges and prospects for large-scale grid storage	status and options for mechanical, thermal, electrochemical, and chemical energy storage	[1]
2018	Journal of Membrane Science	Selectivity of ion exchange membranes: A review	selectivity of ion exchange membranes	[22]
2019	Applied Energy	Recent development of membrane for vanadium redox flow battery applications: A review	research on membranes for VRFB	[23]
2019	Current Opinion in Electrochemistry	Membranes and separators for redox flow batteries	current development trends for membranes and separators for VRFB	[24]
2020	Journal of Energy Storage	Membranes for all vanadium redox flow batteries	different membrane types, membrane performance	[25]
2021	Membranes	Polymer Membranes for all-Vanadium Redox Flow Batteries: A Review	graphical overview of polymer membranes; main polymer, impact on VRFB	this paper

**Table 2:** Overview of commercial membranes for VRFB: (1–9) FumaTech, (10–14) DuPont, (15, 16) Asahi Glass, (17) Solvay.

MS	Membrane	Chem	Operating Mode	CD	CE	VE	EE	Ref.
				mA cm^−2^	%	%	%	
1	FAP-330-PE	AEM	high current density	20–80	95.9	94.4	90.5	[26]
2	FAP-450	AEM	high energy efficiency	20–80	98	90.8	89	[26]
3	FAP-375-PP	AEM	low self-discharging	20–80	99	89.9	89	[26]
4	FS-930	CEM	high current density	20–80	96	94.8	91	[26]
5	F-930-RFD	CEM	high current density	20–80	98.5	92.4	91	[26]
6	F-1075-PK	CEM	low self-discharging	20–80	99.5	90.5	90	[26]
7	F-1850	CEM	low self-discharging	20–80	99.5	83.4	83	[26]
8	VX-20	AEM	low self-discharging	80	99.99	84	84	[32]
9	Fumapem 14,100	CEM	-	40	91.3	90.2	82.4	[33]
10	Vanadion	CEM	high current density	80	88	92	81	[27]
11	Vanadion	CEM	high current density	320	96	76	73	[27]
12	Nafion N117	CEM	high current density	100	96	61	59	[34]
13	Nafion N115	CEM	high current density	80	95	90	86	[27]
14	Nafion 212	CEM	high current density	200	97.6	77.9	76	[35]
15	New Selemion	AEM	-	40	98.6	87.5	86.3	[28]
16	New Selemion CL	AEM	-	60	93.5	87.7	82	[29]
17	Aquivion-E87	CEM	high current density	80	97	86	83	[31]

**Table 3:** The efficiencies of VRFB cells using the listed reference membranes N212, N115 and N117 for investigated current densities.

MS	Membrane	CD	CE	VE	EE	Ref.	MS	Membrane	CD	CE	VE	EE	Ref.
		mA cm^−2^	%	%	%				mA cm^−2^	%	%	%	
18	N212	20	81.2	92	74	[37]	12	N117	100	96	61	59	[34]
18	N212	80	94	70	66	[37]	52	N117	40	90	92	83	[38]
19	N212	25	97	95	92	[39]	52	N117	200	95	66	63	[38]
19	N212	100	97	91	88	[39]	53	N117	40	93.8	90.7	85	[33]
20	N212	50	92	86	79	[40]	54	N117	50	87.6	82.6	72.6	[41]
21	N212	50	92	86	79	[42]	55	N117	50	96.5	91	87.5	[43]
22	N212	30	60	96	58	[44]	56	N117	40	91	93	84	[45]
22	N212	60	78	92.3	71	[44]	56	N117	280	98	54	53	[45]
23	N212	80	--	--	88.8	[46]	57	N117	40	93	89	83	[47]
23	N212	240	--	--	74.8	[46]	58	N117	50	95	87	83	[48]
24	N212	20	--	--	81	[49]	59	N117	30	90	76.6	69	[50]
24	N212	80	--	--	71	[49]	60	N117	20	84	94.1	79	[51]
25	N212	200	91	88	80	[52]	60	N117	80	91	81	73.5	[51]
26	N212	80	94	75	71	[53]	61	N117	20	81	72	86	[54]
27	N212	40	75	95	71	[55]	61	N117	80	95	66	66	[54]
27	N212	200	92	80	72	[55]	62	N117	100	96	63	60.5	[56]
14	N212	100	95.5	91.6	87.5	[35]	63	N117	20	74	81	67	[57]
14	N212	200	97.6	77.9	76	[35]	63	N117	50	83	70	62	[57]
							64	N117	60	91	89	81	[58]
28	N115	80	94.6	86.6	82	[59]	65	N117	40	87	92	80	[60]
29	N115	40	94.5	90.1	85.2	[61]	65	N117	200	93	68	63	[60]
30	N115	20	92.5	92.5	86	[62]	66	N117	30	96.4	90.7	87.4	[63]
30	N115	50	94	82.5	76	[62]	67	N117	20	85	81	68.9	[64]
31	N115	20	94	94	88	[65]	67	N117	80	92	70	64.4	[64]
31	N115	100	97.5	77.5	75	[65]	68	N117	50	89.9	90.8	81.6	[66]
32	N115	40	88	94	82	[45]	69	N117	50	93.8	90.7	85	[67]
32	N115	320	96	56	54	[45]	70	N117	10	72.5	93.8	68	[68]
33	N115	40	94	94.7	89	[69]	70	N117	60	89	75.3	67	[68]
33	N115	200	97.5	73.8	72	[69]	71	N117	40	94	84	79	[70]
34	N115	20	79	95	75	[71]	72	N117	30	90	81	73	[72]
34	N115	80	94	84	79	[71]	72	N117	60	94.5	63	60	[72]
35	N115	20	81	91	73	[73]	73	N117	30	95	76.8	73	[74]
35	N115	80	92	66	61	[73]	73	N117	120	97	64.4	62.5	[74]
36	N115	20	90	81	73	[75]	74	N117	30	90.8	84.8	77	[76]
36	N115	100	93	55	51	[75]	75	N117	60	86.3	80.6	69.6	[77]
37	N115	20	93	87	81	[78]	76	N117	50	93	82.3	77	[79]
37	N115	100	97	62	61	[78]	77	N117	60	92.8	79.6	73.8	[80]
38	N115	80	94.6	82.1	86.8	[81]	78	N117	40	90	92	83	[82]
39	N115	60	91.7	92.3	84.7	[83]	78	N117	200	95	66	63	[82]
40	N115	80	--	--	84.5	[46]	79	N117	80	92	87	80	[84]
40	N115	160	--	--	75.2	[46]	80	N117	20	82	90	74	[85]
41	N115	40	98	72	70	[86]	80	N117	80	92	71	65	[85]
42	N115	80	96.64	86	83	[32]	81	N117	10	72.5	97.5	71	[87]
43	N115	80	94	87	81.8	[88]	81	N117	80	92.5	84	78	[87]
44	N115	50	97	89	86.3	[89]	82	N117	40	89	91	81	[90]
44	N115	150	98	70	68.6	[89]	82	N117	200	94	67	63	[90]
45	N115	50	96	89	86	[91]	83	N117	40	90	92	83	[92]
46	N115	40	93	93	86.5	[93]	83	N117	200	95	67	63.7	[92]
46	N115	120	96	80	76.8	[93]	84	N117	40	95.6	91	86.9	[94]
47	N115	40	96.26	94.3	90.77	[95]	85	N117	40	94	91	86	[96]
47	N115	160	98.11	79.98	78.47	[95]	85	N117	100	96	80	76.8	[96]
48	N115	80	93	88	82	[97]	86	N117	40	95	90	85.5	[98]
49	N115	40	91	93	85	[99]	86	N117	140	96	77	74	[98]
49	N115	160	94	78	73	[99]	87	N117	20	94	95	89.3	[100]
50	N115	50	91.3	91.9	84	[101]	87	N117	80	96	83	79.7	[100]
51	N115	80	92	88	82	[102]	88	N117	40	95.9	89.7	86	[103]
13	N115	80	95	90	86	[27]	89	N117	20	81	87	72	[104]
13	N115	320	97	67	65	[27]	89	N117	80	94	67	65	[104]
							90	N117	20	86.2	90.3	77.8	[105]

**Table 4:** Polymers for the preparation of VRFB membranes.

Polymer	Group	Structure Examples
PFSA, PTFE, PVDF, ETFE	fluoro-carbons	-C-F
Poly (phenylene)	hydro-carbon	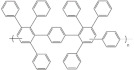
Poly (ether ketone)	hydro-carbon	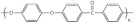
Poly (ether sulfone)	hydro-carbon	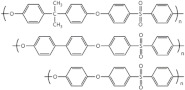
Poly (fluorenyl ether)	hydro-carbon	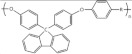
Poly (phenylene ether)	hydro-carbon	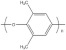
other	hydro-carbon	-
Poly (benzimidazole)	*N*-heterocycles	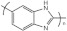
Poly (phthalazinone ether ketone)	*N*-heterocycles	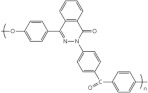
Poly (imide)	*N*-heterocycles	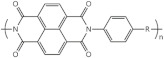

**Table 5:** List of fluoro-carbon membrane samples.

No	Membrane Sample	Membrane		Membrane Properties						VRFB Properties					Reference	
MS	Polymer/Sample Name	Struc	Chem	d	IEC_C_	IEC_A_	WU	D_C_	D_r_	CD	CE	VE	EE	EE_r_	Mem	Pub
				µm	mmol g^−1^	mmol g^−1^	wt.%	cm^2^ min^−1^	%	mA cm^−2^	%	%	%			
91	PTFE/Nafion/P/N	dhe	CEM	45	0.69	-	24.9	4.62 × 10^−8^	0.7	80	96	80	76	1.070	N212	[53]
92	PTFE/P/N/S-7	dhe	CEM	25	-	-	65.5	9 × 10^−8^	0.45	80	94	87	82	1.155	P/N	[115]
93	PTFE/SiO2	sym	-	-	-	-	48	-	-	50	93	86	80	0.930	N115	[91]
94	PTFE/SPEEK/SP60	dhe	CEM	-	-	-	36	-	-	80	93	90	84	1.021	N115	[88]
95	PTFE/ZrP	dhe	CEM	50	1.45	-	-	3.66 × 10^−7^	0.275	40	99	65	64	0.901	N115	[86]
96	PTFE/SE3/P	dhe	CEM	70	-	-	29.8	7.1 × 10^−7^	0.178	100	99	79	78	1.147	N117	[116]
97	PVDF/M7	sym	-	115	-	-	-	-	-	120	94	79	75	-	-	[117]
98	PVDF/M-23-125	asym	-	125	-	-	-	7.9 × 10^−7^	0.664	80	95	83	79	-	-	[118]
99	PVDF/M2	asym	-	105	-	-	-	-	-	80	94	87	82	1.012	N115	[119]
100	PVDF-g-St-co-/AIEM	dho	AIEM	58	1	-	-	1.18 × 10^−7^	0.153	-	-	-	-	-	N117	[120]
101	PVDF g/AIEM	dho	AIEM	25	1.2	-	48	6.9 × 10^−8^	0.087	-	-	-	-	-	N117	[121]
102	PVDF-g-PSSA/22	dho	CEM	115	1.2	-	26.4	2.53 × 10^−7^	0.084	60	91	78	73	1.082	N117	[68]
103	PVDF-g-PSSA-co-PMAc	dho	AIEM	70	1.63	-	-	7.3 × 10^−,^	0.089						N117	[122]
104	PVDF-g-St-co-./AIEM	dho	AIEM	25	0.8	0.7	36	6.7 × 10^−8^	0.084	-	-	-	-	-	N117	[123]
105	PVDF/SiO2-SO3H_42	dhe	CEM	30	-	-	52.1	1.12 × 10^−7^	0.108	60	90.3	83.5	75.6	0.967	N115	[124]
106	PVDF/HA-45	dho	CEM	-	2.67	-	46	2.5 × 10^−7^	-	100	95	84	80	1.08	N117	[125]
107	ETFE-g-PSSA-c- AIEM-II	dho	AIEM	45	1.06	1.24	36.1	2.9 × 10^−9^	0.004	40	96	79	75	1.034	N117	[126]
108	ETFE-g-PSSA	dho	CEM	38	0.88	-	14.7	3.9 × 10^−8^	0.057	-	-	-	-	-	N117	[126]
109	ETFE-g-GMA- DG225	dho	CEM	50	2.4	-	181	-	-	100	87	73	64	1.067	N117	[56]
110	ETFE-g-PDMAEMA/40%	dho	AEM	70	-	1.7	20	3.6 × 10^−8^	0.042	-	-	-	-	-	N117	[127]
111	ETFE-g-poly(VP)	dho	AIEM	25	0.7	-	-	-	-	120	98	73	71	1.044	N117	[128]
112	ETFE-VB-DABACO	dho	AEM	50	-	1.55	38	-	-	-	-	-	-	-	N115	[129]
113	ETFE-VB-DMA	dho	AEM	50	-	1.33	8.8	-	-	-	-	-	-	-	N115	[129]
114	ETFE-VB-TMA	dho	AEM	50	-	1.64	38	-	-	-	-	-	-	-	N115	[129]
115	PFSA AATMS	dhe	AIEM*	125	-	-		8.5 × 10^−7^	0.218	80	96	88	83	1.051	N115	[71]
116	PFSA AATMS (a-SiO2)	dhe	AIEM*	188	1.05	-	35.1	2.32 × 10^−7^	0.268	80	91	72	69	1.062	N117	[64]
117	PFSA CC (CCM)	dhe	CEM	-	-	-	-	-	-	120	95	86	81	1.056	N115	[130]
118	PFSA FC (N/FC-5)	dhe	CEM	-	0.925	-	31	4.1 × 10^−8^	0.5	80	95	78	74	1.250	N117	[112]
119	PFSA GO (GO-0.01)	dhe	CEM	27.75	0.96	-	22.41	-	-	80	92	86	79	1.068	N117	[51]
120	PFSA ND (AMH-3)	dhe	CEM	183	-	-	-	8.64 × 10^−7^	0.281	40	97	85	82	0.965	N117	[47]
121	PFSA Ormosil (N/O)	dhe	CEM	217	0.97	-	23.6	1.85 × 10^−7^	0.050	80	96	84	81	1.210	N117	[131]
122	PFSA [PDDA PSS]5	dhe	AIEM	-	-	-	-	2.85 × 10^−7^	0.095	80	98	73	72	1.091	N117	[85]
123	PFSA [PDDA ZrP]3	dhe	AIEM	-	-	-	8	-	-	50	95	85	81	1.052	N115	[132]
124	PFSA PEI (N/P2.5)	dhe	AIEM*	196	0.89			5.23 × 10^−7^		50	96.2	88.4	85.1	1.001	N117	[67]
125	PFSA PVDF (N/P0.2)	dhe	CEM	100	0.64	-	16.2	-	-	80	94	88	84	1.037	D-520	[133]
126	PFSA (N-sDDS)	dhe	CEM	193	0.92	-	14.3	-	-	70	96	85	81	1.177	N117	[134]
127	PFSA (N/Si/Ti)	dhe	CEM	225	0.95	-	22.5	4.3 × 10^−7^	-	30	95	82	78	1.012	N117	[76]
128	PFSA (N-SiO2)	dhe	CEM	178	-	-	-	8.64 × 10^−7^	-	50	93	91	84	1.000	N117	[135]
129	PFSA SPEEK (N/S)	dhe	CEM	100	1.67	-	-	1.93 × 10^−7^	0.053	50	98	85	83	0.980	N117	[136]
130	PFSA (N-TiO2)	dhe	CEM	90	0.85	-	19.13	6.72 × 10^−6^	0.297	60	89	81	72	1.027	N117	[77]
131	PFSA (CS/PWA)	dhe	AIEM*	58	-	-	-	-	-	60	82	93	76	1.070	N212	[44]
132	PFSA (ZrNT)	dhe	CEM	155	0.927	-	-	3.6 × 10^−9^	0.010	40	98	81	79	1.090	N117	[137]
133	SFPAE 1.8_45	dho	CEM	45	1.8	-	-	-	-	80	99	83	82	-	-	[113]
134	SFPAE 1.8	dho	CEM	60	1.8	-	48	1.16 × 10^−8^	0.219	100	98	92	90	1.047	N212	[39]
135	SFPAE (PVDF-co-/10%)	dhe	CEM	60	1.6	-	35	-	-	100	99	88	88	0.999	SFPAE	[138]

**Table 6:** List of poly(phenylene)-based hydro-carbon membrane samples.

No	Membrane Sample	Membrane		Membrane Properties						VRFB Properties					Reference	
MS	Polymer/Sample Name	Struc	Chem	d	IEC_C_	IEC_A_	WU	D_C_	D_r_	CD	CE	VE	EE	EE_r_	Mem	Pub
				µm	mmol g^−1^	mmol g^−1^	wt.%	cm^2^ min^−1^	%	mA cm^−2^	%	%	%			
136	PP/SDAPP1.8	dho	CEM	41	1.8	-	-	-	-	200	96	88	85	1.181	N117	[139]
137	PP/SDAPP2.2	dho	CEM	50	2.2	-	-	-	-	200	48	88	42	0.583	N117	[139]
138	PP/QDAPP0.8	dho	AEM	54	-	0.8	-	-	-	200	96	88	85	1.181	N117	[139]
139	PP/QDAPP1.2	dho	AEM	54	-	1.2	-	-	-	200	96	88	85	1.181	N117	[139]
140	PP/AMPP11	dho	AEM	80	-	1.1	31.6	3.3 × 10^−9^	0.034	40	99	57	57	0.912	N117	[140]
141	PP/AMPP15	dho	AEM	80	-	1.5	45.2	3.2 × 10^−8^	0.330	40	98	59	58	0.920	N117	[140]
142	PP/QDAPP2	dho	AEM	-	-	0.8	77	1.4 × 10^−6^	0.666	200	97	90	87	1.094	N212	[52]
143	PP/SDAPP (Sample1)	dho	CEM	-	1.4	-	36	4.4 × 10^−7^	0.094	50	99	90	89	1.011	N117	[43]
144	PP/p-TPN1	dho	AEM	35	-	2.15	18	7.4 × 10^−8^	0.018	80	100	85	85	1.06	N212	[141]

**Table 7:** List of ether-ketone-based hydro-carbon membrane samples.

No	Membrane Sample	Membrane		Membrane Properties						VRFB Properties					Reference	
MS	Polymer/Sample Name	Struc	Chem	d	IEC_C_	IEC_A_	WU	D_C_	D_r_	CD	CE	VE	EE	EE_r_	Mem	Pub
				µm	mmol g^−1^	mmol g^−1^	wt.%	cm^2^ min^−1^	%	mA cm^−2^	%	%	%			
145	SPEEK/DS92	dho	CEM	65	2.35	-	88	3.06 × 10^−6^	0.243	200	93	82	77	1.048	N212	[55]
146	SPEEK	dho	CEM	100	1.8	-	-	2.43 × 10^−7^	0.067	50	97	87	85	0.998	N117	[136]
147	SPEEK (N/S)	dhe	CEM	100	1.67	-	-	1.93 × 10^−7^	0.053	50	98	85	83	0.980	N117	[136]
148	SDPEEK (SD4-6-100)	dho	CEM	100	1.2	-	42.5	0.38 × 10^−7^	0.027	50	98	90	88	1.046	N115	[101]
149	SDPEEK (C-SD5-5-50)	dho	CEM	50	1.65	-	29.8	0.48 × 10^−7^	0.034	50	97	93	90	1.024	N115	[101]
150	SPEEK	dho	CEM	35	1.5	-	27	6.88 × 10^−7^	0.433	40	98	91	89	1.145	N112	[33]
151	SPEEK (PANI 80/20)	dhe	CEM	37	1.44	-	21	2.67 × 10^−7^	0.168	40	98	93	91	1.175	N112	[33]
152	SPEEK	dho	CEM	128	-	-	60.6	1.61 × 10^−6^	0.797	40	93	95	88	1.060	N115	[142]
153	SPEEK (SPEEK/RP)	dhe	CEM	108	-	-	51.8	6.9 × 10^−7^	0.342	120	98	81	80	1.060	N115	[142]
154	SPEEK-co-PEEK	dho	CEM	65	2.12	-	55	1.84 × 10^−6^	0.571	80	93	88	82	1.062	N117	[58]
155	SPEEK (S/SBA-20)	dhe	CEM	61	1.72	-	31.8	6.2 × 10^−7^	0.193	80	97	89	86	1.121	N117	[58]
156	SPEEK	dho	CEM	75	2.25	-	86	3.06 × 10^−6^	0.812	200	96	72	70	1.094	N117	[60]
157	SPEEK (SPEEK-15%)	dho	CEM	75	1.9	-	62	1.9 × 10^−6^	0.496	200	98	72	72	1.125	N117	[60]
158	SPEEK/DS 57.99	dho	CEM	195	1.58	-	30.57	2.42 × 10^−8^	0.008	-	-	-	-	-	N117	[143]
159	SPEEK/DS 86.49	dho	CEM	195	2.35	-	83.02	2.28 × 10^−6^	0.740	-	-	-	-	-	N117	[143]
160	SPEEK	dho	CEM	80	1.75	-	32.6	4.2 × 10^−6^	0.636	60	90	84	76	1.133	N117	[72]
161	SPEEK (g-C3N4-1.5)	dhe	CEM	80	0.86	-	20.7	4 × 10^−7^	0.061	60	98	85	84	1.200	N117	[72]
162	SPEEK	dho	CEM	70	-	-	-	1.04 × 10^−7^	0.299	200	97	73	71	1.118	N117	[82]
163	SPEEK (PDA-0.5h)	dho	CEM	70	-	-	-	1.67 × 10^−7^	0.048	200	99	68	67	1.055	N117	[82]
164	SPEEK/S67-DMF	dho	CEM	55	1.97	-	38	2 × 10^−7^	0.053	120	97	83	81	1.095	N117	[84]
165	SPEEK/S87-DMF	dho	CEM	55	2.43	-	81	3.5 × 10^−6^	0.921	120	94	83	77	1.041	N117	[84]
166	SPEEK	dho	CEM	70	-	-	-	-	-	200	100	65	65	1.016	N115	[144]
167	PTFE/SPEEK/PTFE	dhe	CEM	130	-	-	-	-	-	80	99	86	85	-	N115	[144]
168	SPEEK	dho	CEM	200	2	-	50	1.14 × 10^−6^	0.877	-	-	-	-	-	N117	[105]
169	SPEEK/PVDF/TPA	dhe	CEM	200	1.9	-	35.3	5.17 × 10^−7^	0.398	20	93	87	81	1.041	N117	[105]
170	SPEEK	dho	CEM	65	2.24	-	62.6	9 × 10^−7^	0.281	200	98	61	60	0.952	N117	[92]
171	SPEEK (S/A 5%)	dhe	CEM	65	1.99	-	53.5	2 × 10^−7^	0.063	200	97	70	68	1.079	N117	[92]
172	SPEEK (S/S 5%)	dhe	CEM	58	1.92	-	52.7	2 × 10^−7^	0.063	200	98	73	72	1.143	N117	[92]
173	SPEEK (S/T 5%)	dhe	CEM	68	2.07	-	60.6	3.5 × 10^−7^	0.109	200	99	68	67	1.063	N117	[92]
174	C-SPEEK-50	dhe	CEM	90	1.34	-	50	-	-	80	98	87	85	1.040	N115	[59]
175	SPEEK	dho	CEM	80	2.16	-	30.9	1.56 × 10^−6^	0.467	-	-	-	--	-	N117	[145]
176	SPEEK (S/G)	dhe	CEM	90	1.98	-	49.4	8.7 × 10^−7^	0.261	80	98	86	84	1.063	N117	[145]
177	SPEEK	dho	CEM	99	1.49	-	26.7	1.56 × 10^−6^	0.427	-	-	-	-	-	N117	[146]
178	SPEEK (S/OCN-1)	dhe	CEM	100	1.56	-	48.2	9.09 × 10^−7^	0.249	60	98	86	84	1.000	N117	[146]
179	SPEEK	dho	CEM	52	1.85	-	37.1	1.15 × 10^−6^	0.330	200	98	68	67	1.047	N117	[38]
180	SPEEK (S/GO 3)	dhe	CEM	50	2.07	-	44.9	5.9 × 10^−7^	0.171	200	98	72	71	1.109	N117	[38]
181	SPEEK (S/P-0)	dho	CEM	-	1.79	-	38.4	1.12 × 10^−7^	1.172	50	90	91	82	1.007	N117	[66]
182	SPEEK (S/P-3/PEI)	dhe	CEM	-	1.38	-	32.9	4.78 × 10^−8^	0.073	50	97	89	87	1.065	N117	[50]
183	SPEEK	dho	CEM	-	2.03	-	43	4.2 × 10^−6^	0.627	30	96	80	77	1.100	N117	[147]
184	SPEEK (PPD-GO-1)	dhe	CEM	-	1.08	-	22	1.2 × 10^−6^	0.179	30	97	86	83	1.179	N117	[147]
185	SPEEK	dho	CEM	79	2.1	-	47.3	2.5 × 10^−7^	0.104	80	97	76	74	1.021	N117	[40]
186	SPEEK (S/P 15)	dhe	CEM	74	1.79	-	39.6	1 × 10^−7^	0.042	60	98	83	81	1.069	N117	[40]
187	SPEEK	dho	CEM	80	-	-	-	4.03 × 10^−7^	0.490	-	-	-	-	-	N212	[148]
188	SPEEK/SCCT	dhe	CEM	90	-	-	-	3.22 × 10^−7^	0.391	50	99	86	85	1.104	N212	[148]
189	SPEEK (TiO2 5%)	dhe	CEM	-	1.5	-	23	2.45 × 10^−7^	0.076	50	98	82	80.4	1.084	N117	[149]
190	SPEEK (SPEEK-40)	dho	CEM	90	1.45	-	-	0.36 × 10^−7^	0.045	50	99	89	88	1.033	N115	[83]
191	SPEEK (TPA/PP)	dhe	CEM	240	-	-	-	4.78 × 10^−7^	0.581	35.7	96	86	83	1.014	N212	[150]
192	SPEEK (S/PAN 20)	dho	CEM	75	1.78	-	58	11.3 × 10^−7^	0.300	80	96	87	84	1.065	N117	[151]
193	SPEEK (PSP)	dho	CEM	75	0.74	-	7.8	1.37 × 10^−8^	0.050	20	99	76	75	0.935	N117	[152]
194	PEEK-QADMPEK 3	dho	AEM	43	-	1.75	18.8	7.64 × 10^−7^	0.244	80	99	85	84	1.050	N117	[98]
195	QPEK-C-TMA	dho	AEM	40	-	1.4	36	8.2 × 10^−9^	0.028	30	99	83	82	-	N212	[153]
196	SPEKS/sGO 0.5	dhe	CEM	-	0.76	-	31	5 × 10^−8^	0.161	40	99	83.3	82.5	1.12	N212	[154]
197	SPEEK/ZC-GO-2	dhe	AIEM	75	1.87	-	36.5	12.7 × 10^−7^	0.189	50	98.5	92.3	91.4	1.09	N117	[155]
198	S/TPAM-1%	dho	AIEM	108	1.55	-	39.3	3.04 × 10^−7^	0.074	60	97.5	86.6	83.8	1,018	N115	[156]
199	SPEEK/L15	dho	CEM	81	1.11	-	29.62	1.7 × 10^−8^	0.086	120	99.5	83.9	83.5	-	N212	[157]
200	CrSPK45-S	dho	CEM	-	1.67	-	22.16	6.1 × 10^−9^	0.12	80	98	86.7	85	1.08	N117	[158]
201	Q2-ADMPEK-4	dho	AEM	-	-	2.07	24,05	-	-	80	99	88.5	87.6	1.09	N212	[159]
202	CQSPK-6	dho	AEIM		0.95	-	21.6	1.05 × 10^−9^	0.064	60	98.4	82.7	81.4	1.07	N117	[160]
203	SPAEK/Ce2Zr2O7 2%	dhe	CEM	-	1.31	-	52	1.29 × 10^−9^	0.037	40	99.9	82.6	82.1	1.087	N212	[161]

**Table 8:** List of PSU, PPSU and PES-based hydro-carbon membrane samples.

No	Membrane Sample	Membrane		Membrane Properties						VRFB Properties					Reference	
MS	Polymer/Sample Name	Struc	Chem	d	IEC_C_	IEC_A_	WU	D_C_	D_r_	CD	CE	VE	EE	EE_r_	Mem	Pub
				µm	mmol g^−1^	mmol g^−1^	wt.%	cm^2^ min^−1^	%	mA cm^−2^	%	%	%			
204	PSU/PVDF/imi sIPN	dhe	AEM	-	-	-	21	-	-	80	99	85	84	-	-	[162]
205	PSU/CMPSF 72	sym	AEM	45	-	1.51	-	-	-	80	99	87	86	1.048	N115	[102]
206	PSU/ImPSf/SPEEK	dhe	AIEM	65	2.04	-	56	1.5 × 10^−8^	0.071	200	98	79	77	1.069	N212	[55]
207	PSU/PVP 50	dhe	AIEM	50	-	-	-	-	-	100	99	79	78	1.026	N212	[163]
208	PSU/PVP/PS M90	asym	AIEM	130	-	-	-	-	-	80	90	87	78	-	-	[164]
209	PSU/CPSF-Py	sym	AEM	88	-	-	-	-	-	100	97	89	86	1.051	N115	[165]
210	PSU/TMA	dho	AEM	43	-	-	-	2.6 × 10^−8^	-	30	96	88	85	1.012	N212	[166]
211	PSU/PSf-c-PTA-1.4	dho	AEM	-	-	1.7	36.7	2.57 × 10^−7^	0.198	120	98.4	85.7	84.3	1.12	N115	[167]
212	PSU/SPSF-62	dho	CEM	76	1.26	-	24.5	2.94 × 10^−6^	0.438	100	98.8	87.2	86.2	1.14	N117	[168]
213	PSU/SPSF/g-C3N4-1	dhe	AIEM	85	1.11	-	19.9	7 × 10^−7^	-	100	98	89.1	87.3	1.15	N117	[169]
214	PPSU/CMP-2	dho	AEM	42.5	-	1.95	35.3	1.5 × 10^−8^	0.005	80	97	86	83	1.049	N117	[96]
215	PPSU/QA-1.7	dho	AEM	57.5	-	1.7	16	-	-	80	100	75	70	1.106	N212	[114]
216	PPSU/AEM	dho	AEM	50	-	-	-	-	-	60	100	70	70	-	N212	[170]
217	PPSU/S-needle	dho	CEM	115	1.95	-	-	2.07 × 10^−7^	0.161	50	98	85	84	1.005	N117	[48]
218	PPSU/BPSH35	dho	CEM	-	1.52	-	40	1.6 × 10^−9^	0.123	80	99	76	75	1.042	N212	[49]
219	PPSU/S2B2	dho	AIEM*	50	1.2	-	40.2			100	99	79	77	1.305	N117	[34]
220	PES/PVP M3	asym	AEM	115	-	-	-	4 × 10^−6^	-	80	93	85	79	-	-	[171]
221	PES/SPEEK M-35-13	sym	CEM	85	-	-	-	-	-	80	91	86	78	-	-	[172]
222	PES/SPEEK M-35-6	asym	CEM	160	-	-	-	-	-	80	92	85	78	-	-	[173]
223	PES/SPEEK M3	dhe	CEM	65	-	-	-	-	-	80	99	87	86	1.049	N115	[99]
224	PES/SPEEK/FT 10%	asym	CEM	180	-	-	-	3.98 × 10^−6^	-	80	95	86	82	-	N115	[174]
225	PES/SPEEK/N 2.56	asym	CEM	-	-	-	-	-	-	80	99	87	86	1.012	N115	[81]
226	PES/SPEEK/PDDA 7.5	sym	AIEM	132	-	-	-	-	-	80	98	92	90	1.071	N115	[175]
227	PES/SPEEK/PPy	asym	AIEM*	120	-	-	-	-	-	80	97	91	88	1.073	N115	[95]
228	PES/SPEEK/SiO2 M2	dhe	CEM	125	-	-	25	-	-	80	82	87	94	0.989	N115	[93]
229	PES/SPEEK/ZSM-35	asym	CEM	-	-	-	-	-	-	80	98	93	91	1.109	N115	[97]
230	SPES/SPEEK	dhe	CEM	70	0.7	-	21.6	-	-	50	98	86	85	1.090	N212	[176]
231	SPES	dho	CEM	-	2.07	-	121.93	2.5 × 10^−6^	0.809	-	-	-	-	-	-	[143]
232	SPAES S/N	dhe	CEM	-	-	-	-	-	-	200	99	75	74	1.035	N115	[69]
233	SPES (IL-30)	asym	CEM	-	-	-	-	1.41 × 10^−8^	-	140	99	80.13	79.3	0.98	N212	[177]
234	MD2.0-10	dhe	AEIM	-	-	-	-	1.7 × 10^−7^	0.134	80	99.3	82.6	82	1.025	N115	[178]

**Table 9:** List of fluorenyl-ether-based hydro-carbon membrane samples.

No	Membrane Sample	Membrane		Membrane Properties						VRFB Properties					Reference	
MS	Polymer/Sample Name	Struc	Chem	d	IEC_C_	IEC_A_	WU	D_C_	D_r_	CD	CE	VE	EE	EE_r_	Mem	Pub
				µm	mmol g^−1^	mmol g^−1^	wt.%	cm^2^ min^−1^	%	mA cm^−2^	%	%	%			
235	QA-PFE	dho	AEM	56	-	2.0	-	-	-	60	100	70	70	1.0	N212	[37]
236	SPECIAL	dho	CEM	112.5	1.92	-	29	-	0.25	50	66.2	73.7	48.8	-	N117	[179]
237	F-SPFEK	dho	CEM	112.5	1.88	-	33	-	0.75	50	76.1	80.3	61.1	-	N117	[179]
238	F-SPFEK-APTES	dho	CEM	112.5	1.75	-	26	-	0.50	50	80.4	79.7	64.1	-	N117	[179]
239	SPECIAL	dho	CEM	161	1.92	-	39	-	0.21	60	87.5	-	-	-	N117	[180]
240	SPFEK/3%SIO2	dhe	CEM	155	1.83	-	36.6	-	0.29	60	87.5	-	-	-	N117	[180]
241	SPECIAL	dho	CEM	180	1.92	-	27.8	9.85 × 10^−7^	0.40	40	80.3	64.6	51.9	-	N117	[180]
242	SPECIAL	dho	CEM	-	1.87	-	-	-	-	-	-	-	-	-	N117	[181]
243	SPFEK/5ZrPSPP	dhe	CEM	-	1.96	-	-	-	-	50	89	60.7	54	-	N117	[181]
244	SPFEK-[PDDA/PSS]n2	dhe	AIEM	130	-	-	-	-	-	-	-	-	-	-	N117	[182]
245	SPFEK 20.7 [PDA/PSS]2	sym	AIEM	151	-	-	-	5.92 × 10^−7^	0.36	-	-	-	-	-	N115	[183]
246	SPECIAL	dho	CEM	160	1.57	-	36.5	2.67 × 10^−7^	0.13	-	-	-	-	-	N115	[184]
247	SPFEKA 10%.	dho	AIEM	160	1.52	-	30.6	1.56 × 10^−7^	0.08	-	-	-	-	-	N115	[184]
248	SPFEKA-20%	dho	AIEM	160	1.47	-	30.9	0.88 × 10^−7^	0.04	-	-	-	-	-	N115	[184]
249	HSPAEK	dho	CEM	60	1.72	-	38.5	5.5 × 10^−7^	0.16	80	98	85	83	1.05	N117	[90]

**Table 10:** List of poly(phenylene ether)-based hydro-carbon membrane samples.

No	Membrane Sample	Membrane		Membrane Properties						VRFB Properties					Reference	
MS	Polymer/Sample Name	Struc	Chem	d	IEC_C_	IEC_A_	WU	D_C_	D_r_	CD	CE	VE	EE	EE_r_	Mem	Pub
				µm	mmol g^−1^	mmol g^−1^	wt.%	cm^2^ min^−1^	%	mA cm^−2^	%	%	%			
250	BrPPO/Py-42	dho	AEM	50	-	-	13	0.12 × 10^−7^	0.02	100	98.1	84	82	0.932	N212	[35]
251	BrPPO/Py-56	dho	AEM	50	-	-	18	0.2 × 10^−7^	0.03	100	97.7	94	92	1.045	N212	[35]
252	BrPPO/Py-70	dho	AEM	50	-	-	21	0.36 × 10^−7^	0.05	100	96.7	90	87	0.989	N212	[35]
253	SPPO-GO	dhe	CEM	-	1.17	-	16.3	1.1 × 10^−8^	0.05	40	98	71	69.6	-	N212	[185]

**Table 11:** List of other hydro-carbon-based membrane samples.

No	Membrane Sample	Membrane		Membrane Properties						VRFB Properties					Reference	
MS	Polymer/Sample Name	Struc	Chem	d	IEC_C_	IEC_A_	WU	D_C_	D_r_	CD	CE	VE	EE	EE_r_	Mem	Pub
				µm	mmol g^−1^	mmol g^−1^	wt.%	cm^2^ min^−1^	%	mA cm^−2^	%	%	%			
254	QPTM	dho	AEM	89	-	2.08	8.8	1.19 × 10^−7^	0.034	50	100	75	75	1.033	N117	[41]
255	QVTD 2-3	dho	AEM	-	-	-	-	-	-	40	95	80	75	-	-	[186]
256	VBC AVSH-3	dho	AEM	-	-	-	-	-	-	40	95	75	75	-	-	[187]
257	PVC/silica	sym	-	390	-	-	-	-	-	40	89	88	78	0.907	N115	[188]
258	Si-PWA/PVA	dhe	CEM	125	1.2	-	-	6.9 × 10^−8^	0.119	-	-	-	-	-	N115	[189]
259	DHIM-375	dho	CEM	100	0.69	-	31	1.56 × 10^−7^	0.052	20	91	80	72	-	N117	[190]
260	ZPPT-6	dho	AIEM	80	-	1.22	30	-	-	50	98	80	78	1.05	N117	[57]
261	PIM-1	asym	-	“0.75”	-	-	-	-	-	20	97.1	92.5	89.9	1.2	N112-	[191]

**Table 12:** List of PBI-based *N*-heterocycle membrane samples.

No	Membrane Sample	Membrane		Membrane Properties						VRFB Properties					Reference	
MS	Polymer/Sample Name	Struc	Chem	d	IEC_C_	IEC_A_	WU	D_C_	D_r_	CD	CE	VE	EE	EE_r_	Mem	Pub
				µm	mmol g^−1^	mmol g^−1^	wt.%	cm^2^ min^−1^	%	mA cm^−2^	%	%	%			
262	mPBI	dho	AEM	27	-	-	-	2.86 × 10^−9^	0.008	50	99.5	80.4	80	0.941	N115	[65]
263	BlpPBI	dho	AEM	27	-	-	-	3.45 × 10^−8^	0.099	50	99	88.4	87.5	1.029	N115	[65]
264	mPBI-15	dho	AEM	15	-	-	-	0	-	120	99.8	68	67.9	0.893	N212	[192]
265	mPBI-35	dho	AEM	35	-	-	-	0	-	120	99	53	52.5	0.691	N212	[192]
266	p-PBI	sym	AEM	87	-	-	-	4.5 × 10^−8^	0.028	40	99	88	87	1.145	N112	[193]
267	PBI-0%	dho	AEM	16	-	-	-	-	-	40	95	50	47	0.595	N117	[70]
268	PBI 10%.	sym	AEM	45	-	-	-	1.17 × 10^−7^	-	40	99	79	78	0.987	N117	[70]
269	PBI-200	asym	AEM	100	-	-	-	3 × 10^−9^	-	80	99	83	82	1.206	N211	[194]
270	PBI-O/PBI-34	sym	AEM	34	-	-	-	-	-	80	99	91	90	1.092	N115	[195]
271	CSOPBI-NH2 (9/1)	dho	AIEM*	55	0.24	-	47.4	6 × 10^−9^	0.001	60	98	86	84	1.024	N117	[87]
272	-6F-co-10%BI	dho	AIEM*	64	1.56	-	-	2.24 × 10^−11^	-	30	99	91	90	1.027	N117	[63]
273	-6F-co-10%BI-cld	dho	AIEM*	65	1.50	-	-	1.28 × 10^−11^	-	30	99	90	89	1.018	N117	[63]
274	FPAE-6F-PBI S1B1	dhe	AIEM*	50	1.02	-	23.8	-	-	100	100	64	64	1.085	N117	[34]
275	B20N10	dho	AEIM	30	-	-	23.6	1.95 × 10^−9^	0.006	80	100	82.2	82.2	1.068	N115	[196]
276	CPBI-70-NMG	dho	AEM	-	-	-	-	-	-	120	99	86	85.3	1.036	N212	[197]
277	0,7µm PBI	asym	AEM	30	-	-	-	-	-	120	98.5	85	83	1.034	N212	[198]
278	PWN/F6PBI(9/1)	dho	AIEM	45	1.51	-	-	-	-	40	99	81	81	1	N212	[199]
279	PVDF-PBI	asym	AEM	-	-	-	-	-	-	60	98.4	83.3	82	1.03	N117	[200]
280	sPBI	dho	AEIM	220.2	-	-	58.1	5.74 × 10^−7^	-	242	93	86	81	-	N212	[201]
281	PE/PBI	dhe	AEM	25	-	-	20.9	5.04 × 10^−7^	0.346	200	99	81	80.11	1.03	N212	[202]

**Table 13:** List of poly(phthalazinone ether)-based *N*-heterocycle membrane samples.

No	Membrane Sample	Membrane		Membrane Properties						VRFB Properties					Reference	
MS	Polymer/Sample Name	Struc	Chem	d	IEC_C_	IEC_A_	WU	D_C_	D_r_	CD	CE	VE	EE	EE_r_	Mem	Pub
				µm	mmol g^−1^	mmol g^−1^	wt.%	cm^2^ min^−1^	%	mA cm^−2^	%	%	%			
282	PyPPEK-2	dho	AEM	-	-	1.4	17.4	-	-	60	99	85	84	1.000	N117	[203]
283	QAPPEK-2	dho	AEM	-	-	1.5	21	-	-	60	99	83	82	0.964	N117	[203]
284	QAPPEKK	dho	AEM	-	-	1.56	-	-	-	40	99	89	88	1.026	N117	[103]
285	PyPPEKK-4	dho	AEM	42	-	1.55	16.5	-	-	40	98	90	89	1.034	N117	[103]
286	QBPPEK 80	dho	AEM	47	-	1.53	23.8	-	-	40	99	89	88	1.023	N117	[204]
287	QAPPEKK-4	dho	AEM	50	-	1.56	20.8	-	-	20	98	93	91.3	1.016	N117	[100]
288	SPEC	dho	CEM	200	1.272	-	32.34	2.77 × 10^−7^	0.024	60	99	69	68	0.919	N117	[80]
289	SPPEK TPA-17	dhe	CEM	200	1.142	-	33.28	5.75 × 10^−7^	0.049	60	99	76	75	1.010	N117	[80]
290	SPPEK/WO3	dhe	CEM	210	-	-	48.15	3.97 × 10^−7^	0.034	50	98	80	79	1.032	N117	[79]
291	SPPEK P-90	dho	CEM	53	1.51	-	23.2	-	-	40	98	89	87	1.023	N115	[61]
292	SPPES/SP-02	dho	CEM	260	1.42	-	17.42	1.24 × 10^−7^	0.055	40	93	73	68	1.004	N117	[205]

**Table 14:** List of poly(phthalazinone ether)-based *N*-heterocycle membrane samples.

No	Membrane Sample	Membrane		Membrane Properties						VRFB Properties					Reference	
MS	Polymer/Sample Name	Struc	Chem	d	IEC_C_	IEC_A_	WU	D_C_	D_r_	CD	CE	VE	EE	EE_r_	Mem	Pub
				µm	mmol g^−1^	mmol g^−1^	wt.%	cm^2^ min^−1^	%	mA cm^−2^	%	%	%			
293	SPI (ODA)	dho	CEM	60	1.2	-	21.93	2.17 × 10^−7^	0.127	-	-	-	-	-	N117	[74]
294	bSPI (APABI)	dho	AIEM*	54	1.3	-	28.80	1.75 × 10^−7^	0.102	-	-	-	-	-	N117	[74]
295	bSPI(MDA)	dho	CEM	55	1.37	-	34.88	4.43 × 10^−7^	0.259	-	-	-	-	-	N117	[74]
296	bSPI(BAPP)	dho	CEM	57	1.14	-	20.03	2.89 × 10^−7^	0.169	120	99	64	63	1.018	N117	[74]
297	SPI(H)	dho	CEM	50	1.65	-	-	-	-	50	95	74	70	-	-	[206]
298	s-FSPI	dho	CEM	-	1.50	-	17.78	7.4 × 10^−8^	0.055	60	100	77	77	1.160	N115	[207]
299	6F-SPI-50	dho	CEM	-	-	-	-	2.27 × 10^−7^	0.172	60	99.5	72.4	72	1.091	N115	[75]
300	SPI	dho	CEM	50	1.58	-	25.7	2.25 × 10^−7^	0.165	60	98	78	76	1.086	N115	[78]
301	6F-s-bSPI	dho	CEM	35	1.54	-	16.5	1.18 × 10^−7^	0.087	60	100	79	79	1.129	N115	[78]
302	SPI	dho	CEM	69	1.75	-	39.92	1.89 × 10^−7^	0.111	70	93	70	65	0.956	N117	[104]
303	SPI/AlOOH-10	dhe	CEM	58	0.95	-	48.59	1.14 × 10^−7^	0.067	70	96	73	70	1.029	N117	[104]
304	SPI(APABI)	dho	AIEM*	65	1.24	-	22.79	7.2 × 10^−8^	0.042	30	100	77	77	1.069	N117	[208]
305	SPI(BAPP)	dho	CEM	62	1.49	-	27.08	4.8 × 10^−8^	0.028	30	100	79	79	1.097	N117	[208]
306	SPI(MDA)	dho	CEM	64	1.48	-	26.94	2.36 × 10^−7^	0.138	30	98	72	71	0.986	N117	[208]
307	SPI	dho	CEM	45	1.61	-	41.40	1.89 × 10^−7^	0.123	40	94	92	87	0.998	N117	[94]
308	SPI/CS	dhe	AIEM*	50	1.65	-	28.66	1.12 × 10^−7^	0.073	40	98	91	89	1.020	N117	[94]
309	SPI	dho	CEM	65	1.58	-	37.14	2.37 × 10^−7^	0.139	-	-	-	-	-	N117	[209]
310	SPI/MoS2	dhe	CEM	65	1.25	-	29.36	2.02 × 10^−7^	0.119	80	95	65	62	1.016	N117	[209]
311	SPI/s-MoS2	dhe	CEM	66	1.70	-	32.20	1.23 × 10^−7^	0.072	80	96	66	63	1.033	N117	[209]
312	SPI	dho	CEM	50	1.25	-	54.7	2.6 × 10^−6^	0.388	40	89	77	69	1.045	N117	[210]
313	SPI/PEI-GO-2	dhe	AIEM*	50	1.16	-	44.2	7 × 10^−7^	0.104	40	95	82	77	1.167	N117	[210]
314	SPI	dho	CEM	55	1.40	-	38.46	1.9 × 10^−7^	0.111	-	-	-	-	-	N117	[54]
315	SPI/TiO2	dhe	CEM	49	1.24	-	32.94	2.02 × 10^−7^	0.118	70	97	72	69	1.022	N117	[54]
316	SPI	dho	CEM	50	1.25	-	54.7	2.6 × 10^−6^	0.388	80	94	65	63	1.050	N117	[211]
317	SPI/ZGO-4	dhe	AIEM*	50	1.52	-	63.1	1.2 × 10^−6^	0.179	40	93	83	77	1.132	N117	[211]
318	SPI	dho	CEM	66	1.51	-	37.52	2.37 × 10^−7^	0.139	70	93	71	66	1.048	N117	[212]
319	SPI/ZrO2-15	dhe	CEM	74	0.93	-	53.19	2.18 × 10^−7^	0.127	70	97	70	68	1.079	N117	[212]
320	SPI	dho	CEM	150	0.40	-	-	-	-	100	98	73	72	1.220	N117	[213]

**Table 15:** High energy efficiency ratios.

MS	Polymer Used	EE_r_	CD	D_r_	d	WU	IEC	Ref.
		-	mA cm^−2^	-	µm	wt.%	mmol g^−1^	
219	PPSU	1.305	100	-	50	-	1.2	[34]
118	PFSA	1.250	80	0.5	-	31	0.925	[112]
320	PI	1.220	100	-	150	-	0.4	[213]
121	PFSA	1.210	80	0.05	217	23.6	0.97	[131]
269	PBI	1.206	80	-	-	-	-	[194]
261	other	1.200	20	-	-	-	-	[191]
161	SPEEK	1.200	60	0.061	80	20.7	0.86	[72]
136	sDAPP	1.181	200	-	41	-	1.8	[139]
139	qDAPP	1.181	200	-	54	-	1.2	[139]
184	SPEEK	1.179	30	0.179	-	22	1.08	[147]
126	PFSA	1.177	70	-	193	14.3	0.92	[134]
151	SPEEK	1.175	40	0.168	37	21	1.44	[33]
313	PI	1.167	40	0.104	50	44.2	1.16	[210]
298	PI	1.160	60	0.055	-	17.8	1.5	[207]
92	PTFE	1.155	80	0.45	25	65.5	-	[115]

**Table 16:** Cycle stability of published membrane samples.

Polymer	MS	Cycles	mL | mA cm^−^^2^	Ref.	Polymer	MS	Cycles	mL | mA cm^−^^2^	Ref.
PTFE	94	700	- | 80	[88]	PES	224	70	40 | 80	[174]
PVDF	97	50	30 | 80	[117]		230	100	80 | 50	[176]
	98	1000	30 | 80	[118]	PPE	251	500	25 | 200	[35]
	99	300	30 | 80	[119]	other	254	4000	- | 50	[41]
	102	230	40 | 60	[68]		255	150	3 | 40	[186]
PFSA	116	150	60 | 80	[64]		256	500	3 | 40	[187]
	117	300	- | 120	[130]		259	120	3 | 20	[190]
DAPP	143	400	100 | 50	[43]	PBI	262	200	100 | 50	[65]
PEEK	155	120	50 | 60	[58]		270	13000	60 | 80–120	[195]
	157	100	50 | 80	[60]		271	300	10 | 60	[87]
	161	300	10 | 30	[72]		272	220	20| 30	[63]
	167	1000	- | 80	[144]	PPEK	290	100	120 | 50	[79]
	174	180	30 | 80	[59]		291	100	30 | 60	[61]
	176	500	50 | 80	[145]	PI	296	500	30 | 30–120	[74]
	180	50	50 | 60	[38]		297	750	30 | 50	[206]
	194	100	30 | 80	[98]		298	100	- | 60	[207]
PSU	204	900	- | 80	[162]		301	100	60 | 60	[78]
	205	6000	100 | 120	[102]		311	500	30 | 25–70	[209]
	207	500	50 | 80	[163]		313	100	8 | 40	[210]
	208	300	30 | 80	[164]		317	100	8 | 40	[211]
PES	220	150	30 | 80	[171]	Nafion	32	200	50 | 160	[45]
	221	250	60 | 80	[172]		56	200	50 | 160	[45]

**Table 17:** Published low-cost membranes.

MS	Membrane Polymer	Ref.	MS	Membrane Polymer	Ref.
224	PES	[174]	129	PFSA	[136]
151	PEEK	[33]	167	PEEK/PTFE	[144]
263	PBI	[65]	93	PTFE	[91]
191	PEEK	[150]	98	PVDF	[118]
217	PPSU	[48]	251	PPE	[35]
297	PI	[206]	207	PSU	[163]
266	PBI	[193]	286	PPEK	[204]
243	PF	[181]	209	PSU	[165]
258	Other	[189]	10	PFSA	[27]

**Table 18:** Evaluation matrix of polymer membranes (+ less good, ++ good, +++ best).

Membrane Chemistry and Structure	Efficiency			Membrane Material and Structure	Cost
	CE	VE	EE		
CEM	+	+++	++	fluoro-carbon	+
AEM	+++	+	++	hydro-carbon	+++
AIEM	++	++	+++	N-heterocycle	++
dense	+++	++	+++	dense	+++
sym	+	+++	++	sym	++
asym	++	++	++	asym	++

## Data Availability

The data presented in this study are cited (reference numbers).

## Supplementary Materials

https://www.mdpi.com/2077-0375/11/3/214/s1, Figure S1: The water uptake of developed membranes in recent years: (a) fluoro-carbons, (b) hydro-carbons and (c) *N*-heterocycles. Figure S2: The diffusion coefficient ratio of developed membranes in recent years: (a) fluoro-carbons, (b) hydro-carbons and (c) *N*-heterocycles. Figure S3: The anion exchange capacity of developed membranes in recent years: (a) fluoro-carbons, (b) hydro-carbons and (c) *N*-heterocycles.

## Abbreviations

AEM	anion exchange membrane
AEM*	anion exchange membrane (acidic environment)
AIEM	amphoteric ion exchange membrane
AIEM*	amphoteric ion exchange membrane (acidic environment)
asym	asymmetric
CD	current density
CE_H_	coulombic efficiency (≥ 100 mA cm^−2^)
CE_L_	coulombic efficiency (< 100 mA cm^−2^)
CEM	cation exchange membrane
chem	chemistry
CL	cross-linked
d	membrane thickness
D_c_	diffusion coefficient
D_r_	diffusion coefficient ratio
DAPP	diels-Alder Poly(phenylene)
dhe	dense and heterogeneous
dho	dense and homogeneous
DOE	U.S. Department of Energy
EE	energy efficiency (charge-discharge)
EE_r_	energy efficiency ratio
EE_H_	energy efficiency (≥ 100 mA cm^−2^)
EE_L_	energy efficiency (< 100 mA cm^−2^)
ETFE	poly(ethylene-tetrafluoroethylene)
FPAE	fluorinated poly(arylene ether)
IEC	ion exchange capacity
Mem	membrane
MS	membrane sample
PA	poly(amide)
PBI	poly(benzimidazole)
PEEK	poly(ether ether ketone)
PEK	poly(ether ketones)
PES	poly(ether sulfone)
PF	poly(fluorenyle)
PFSA	perfluorosulfonic acid
PI	poly(imide)
PPE	poly(phenylene ether)
PPEK	poly(phthalazinone ether ketones)
PPh	poly(phenylene)
PPSU	poly(phenyl sulfones)
PS	poly(styrene)
PSU	poly(sulfones)
PTFE	poly(tetrafluoroethylene)
pub	publication
PVA	poly(vinyl alcohol)
PVC	poly(vinyl chloride)
PVDF	poly(vinylidene fluoride)
Ref	reference
struc	structure
sym	symmertric
V	vanadium
VE_H_	voltage efficiency (≥ 100 mA cm^−2^)
VE_L_	voltage efficiency (< 100 mA cm^−2^)
VRFB	all vanadium redox flow battery
WU	water uptake
