# Supplementary material for: Polymer Membranes for All-Vanadium Redox Flow Batteries: A Review
Source: Membranes (Basel). 2021 Mar 18;11(3):214. doi: 10.3390/membranes11030214 (PMC8003036; doi:10.3390/membranes11030214)
Supplement: Supplementary file 1 [file membranes-11-00214-s001.pdf]

membranes

Review

Polymer Membranes for all-Vanadium Redox Flow Batteries: A Review

Dennis Düerkop [1,*], Hartmut Widdecke [1], Carsten Schilde [2], Ulrich Kunz [3] and Achim Schmiemann [1]

[1] Institute of Recycling, Ostfalia University of Applied Sciences, Robert-Koch-Platz 8a, 38440 Wolfsburg, Germany; h.widdecke@ostfalia.de (H.W.); a.schmiemann@ostfalia.de (A.S.)

[2] Institute of Particle Technology, Braunschweig University of Technology, Volkmaroder Straße 5, 38100 Braunschweig, Germany; c.schilde@tu-bs.de

[3] Institute of Chemical and Electrochemical Process Engineering, Clausthal University of Technology, Leibnizstraße 17, 38678 Clausthal-Zellerfeld, Germany; kunz@icvt.tu-clausthal.de

* Correspondence: den.dueerkop@ostfalia.de

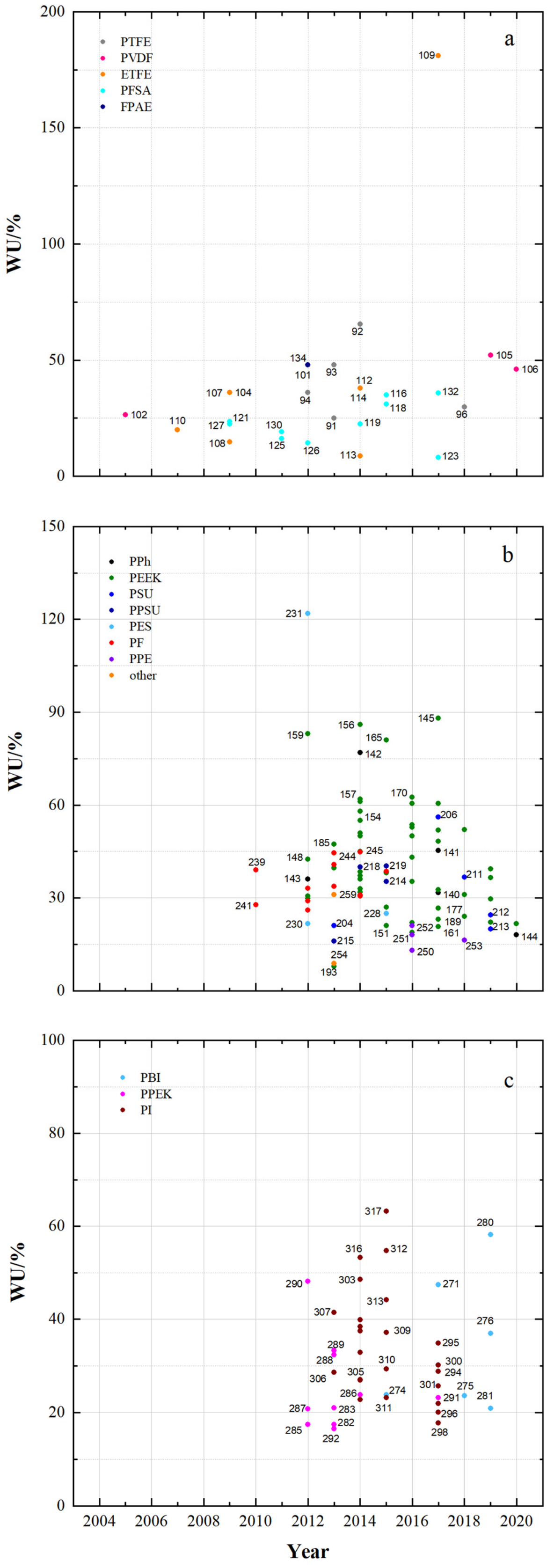

Figure S1: The water uptake of developed membranes in recent years: (a) fluoro-carbons, (b) hydro-carbons and (c) *N*-heterocycles.

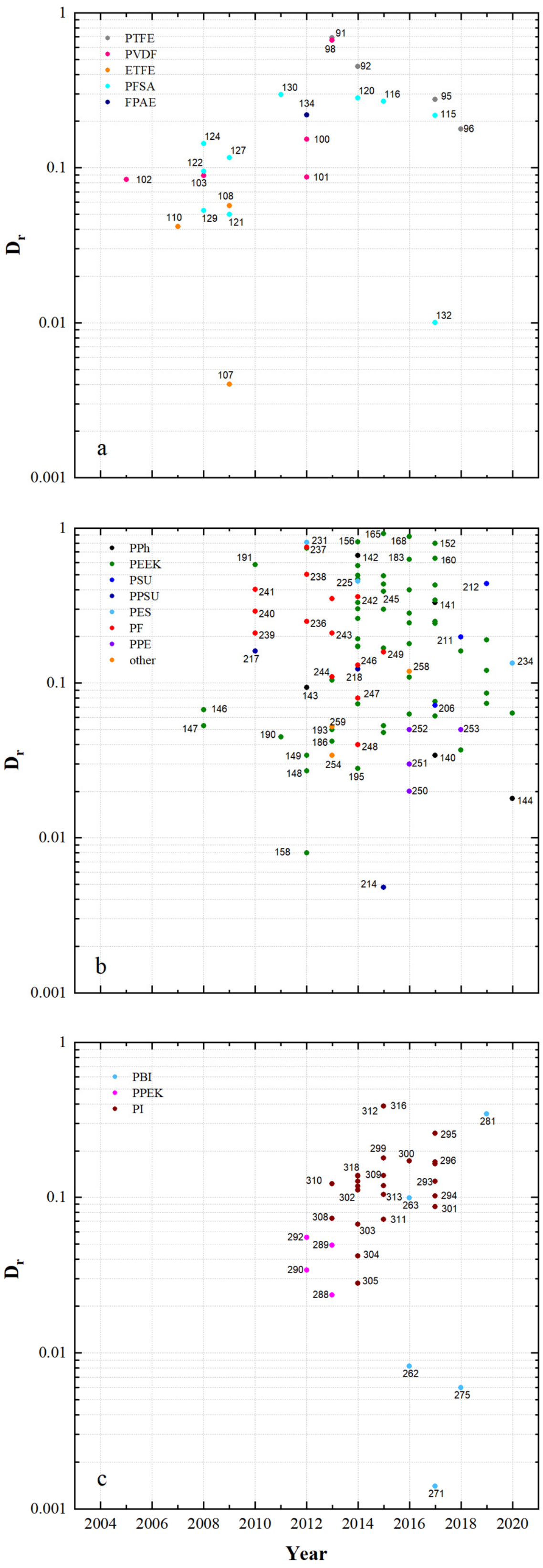

Figure S2: The diffusion coefficient ratio of developed membranes in recent years: (a) fluoro-carbons, (b) hydro-carbons and (c) *N*-heterocycles.

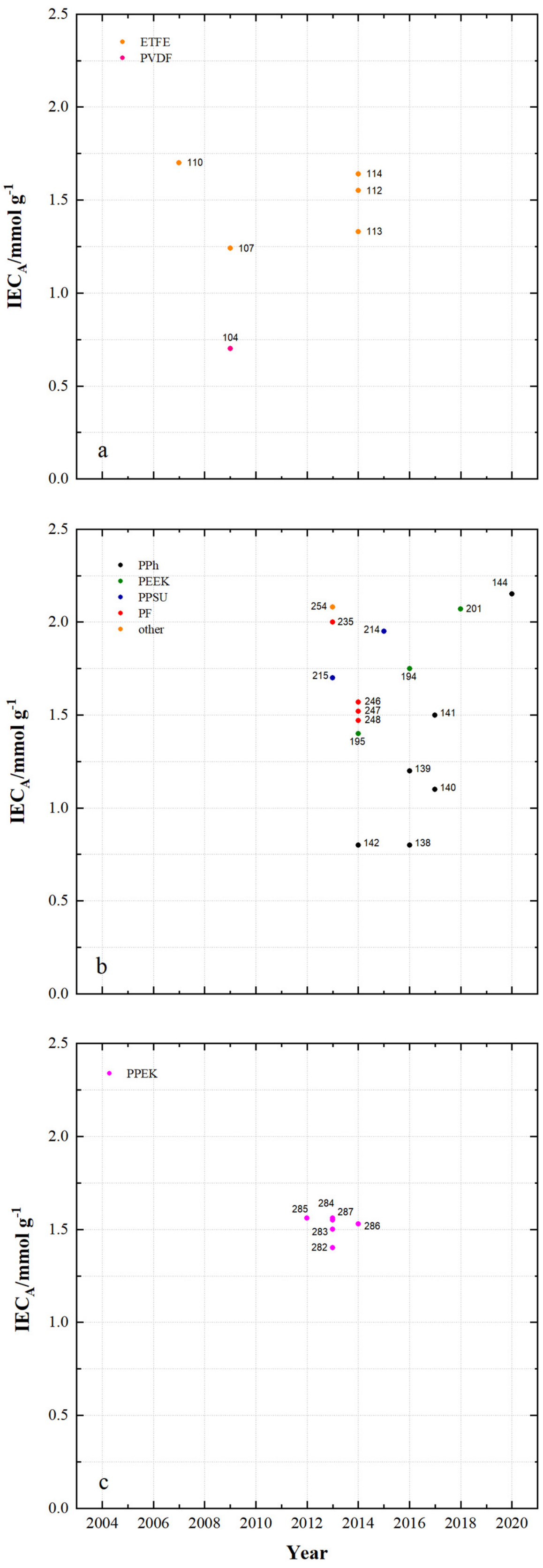

Figure S3: The anion exchange capacity of developed membranes in recent years: (a) fluoro-carbons, (b) hydro-carbons and (c) *N*-heterocycles.
